# Cell membrane-coated nanoparticles against non-alcoholic fatty liver disease via regulating endoplasmic reticulum stress

**DOI:** 10.1093/rb/rbag084

**Published:** 2026-04-30

**Authors:** Weiwei Li, Long Yang, Jiao Lu, Shouhui Yi, Longpo Cao, Kai Lei, Ziyi Sheng, Zuojin Liu

**Affiliations:** Department of Hepatobiliary Surgery, The Second Affiliated Hospital of Chongqing Medical University, Chongqing, 400010, China; Department of Hepatobiliary Surgery, The Second Affiliated Hospital of Chongqing Medical University, Chongqing, 400010, China; Department of Hepatobiliary Surgery, The Second Affiliated Hospital of Chongqing Medical University, Chongqing, 400010, China; Department of Hepatobiliary Surgery, The Second Affiliated Hospital of Chongqing Medical University, Chongqing, 400010, China; Department of Cancer Center, The Second Affiliated Hospital of Chongqing Medical University, Chongqing, 400010, China; College of Biomedical Engineering, Chongqing Medical University, Chongqing, 400016, China; Department of Hepatobiliary Surgery, The Second Affiliated Hospital of Chongqing Medical University, Chongqing, 400010, China; Department of Hepatobiliary Surgery, The Second Affiliated Hospital of Chongqing Medical University, Chongqing, 400010, China; Department of Hepatobiliary Surgery, The Second Affiliated Hospital of Chongqing Medical University, Chongqing, 400010, China

**Keywords:** non-alcoholic fatty liver disease, endoplasmic reticulum stress, cell membrane-coated nanoparticles, GalNAc

## Abstract

Non-alcoholic fatty liver disease (NAFLD) has emerged as a public health challenge, yet the available therapeutic options remain limited. Evidence suggests that NAFLD may advance to more serious conditions, including steatohepatitis or cirrhosis, which are associated with poor outcomes. Current drug therapies exhibit insufficient therapeutic efficacy due to short half-life and premature clearance. In this work, we elucidate the critical impact of endoplasmic reticulum stress (ERS) on inflammatory process associated with NAFLD using bioinformatics tools. By analysing single-cell RNA sequencing data from an animal model of NAFLD, we identified hepatocytes as the primary cell type implicated in ERS. Melatonin (Mel), a compound known for its antioxidative properties, also modulates the ERS pathway, suggesting its potential to slow NAFLD progression. To exploit this therapeutic potential, we developed a nanoparticle (NP), GalNAc-MM@PLGA/Mel, which incorporates engineered macrophage membranes to specifically target hepatocytes and mitigate ERS. The addition of GalNAc moieties on the membrane significantly enhanced the ability of NPs to specifically bind to hepatocytes. Moreover, Mel, delivered through this platform, effectively reduced inflammation and lipid accumulation by inhibiting both the ERS pathway and its downstream NF-κB signaling cascade. Consequently, this engineered NP-based strategy offers a promising, safe and cost-efficient approach to managing NAFLD.

## Graphical Abstract

**Graphical Abstract:**
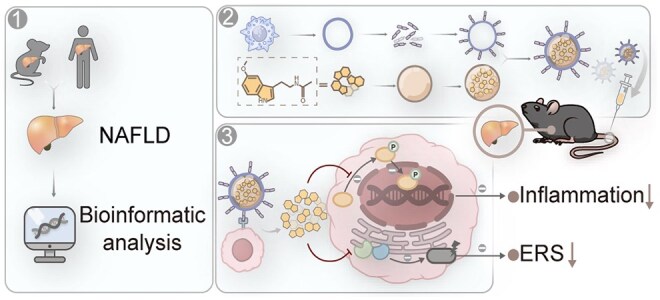

## Introduction

Non-alcoholic fatty liver disease (NAFLD) is one of the most common chronic liver diseases [1]. Evidence suggests that NAFLD may advance to more serious conditions, including steatohepatitis or cirrhosis, which are associated with poor long-term outcomes [2]. Endoplasmic reticulum stress (ERS) is notably prevalent within the NAFLD and is pivotal in the advancement of the disease [3]. ERS triggers pathways involved in lipid synthesis, resulting in the buildup of fatty deposits [4, 5]. This accumulation, in turn, activates inflammatory pathways, particularly the NF-κB signaling pathway [6]. These interconnected processes generate a persistent inflammatory state, which exacerbates injury, further promoting disease progression [7]. At present, effective clinical treatments for NAFLD remain lacking [8].

Melatonin (Mel), a neurohormone synthesized by the pineal gland, is commonly acknowledged for its antioxidant properties, particularly its ability to scavenge free radicals [9]. Previous studies have demonstrated the efficacy of Mel in mitigating NAFLD in animal models, largely due to its antioxidative actions [10]. Moreover, excessive ROS production has been identified as an important upstream trigger of ERS and unfolded protein response activation [11]. Additionally, Mel has been found to specifically inhibit Activating Transcription Factor 6 (ATF6), a key receptor involved in ERS [12]. These mechanisms may allow Mel to modulate ERS and the unfolded protein response [13], underscoring its potential as a therapeutic agent for addressing hepatic steatosis and inflammation associated with NAFLD [14]. However, the short half-life of Mel in circulation significantly hinders its effectiveness [15]. To enhance its therapeutic potential, it is common for researchers to give elevated intravenous dosages (e.g. 20 mg/kg), though this approach carries a greater risk of adverse effects, such as arrhythmias [16]. In short, the low solubility, brief circulation time and potential side effects of Mel have limited its effectiveness as a therapeutic agent. Polylactic acid-hydroxyacetic acid copolymer (PLGA), composed of lactic acid and hydroxyacetic acid, has been granted approval by the FDA for medical application [17]. To deliver Mel into the liver and ensure sustained release, PLGA was selected as the drug carrier due to its superior bio-compatibility [18] and controllable release behavior [19]. Recently, nanoparticles (NPs) coated with natural cell membranes have been introduced as an innovative approach to enhance the properties of synthetic NPs [20, 21]. These biomimetic particles not only preserve the key functional characteristics of their originating cell membranes but also significantly enhance the cycling stability [22], immune evasion [23] and bio-compatibility [24] of the NPs. Various forms of cell membrane-coated NPs have been successfully engineered to reduce lipid accumulation, inhibit inflammatory responses or correct mitochondrial dysfunction in the treatment of NAFLD [25, 26]. Among these, NPs coated with macrophage membranes have gained attention due to their capability to evade phagocytosis by the mononuclear phagocyte system *in vivo* [27]. Compared to traditional cell membrane-coated NPs, engineered cell membrane-coated NPs offer greater advantages, including superior bio-compatibility and the potential for customization to meet specific therapeutic objectives [28].

**Figure 1:**
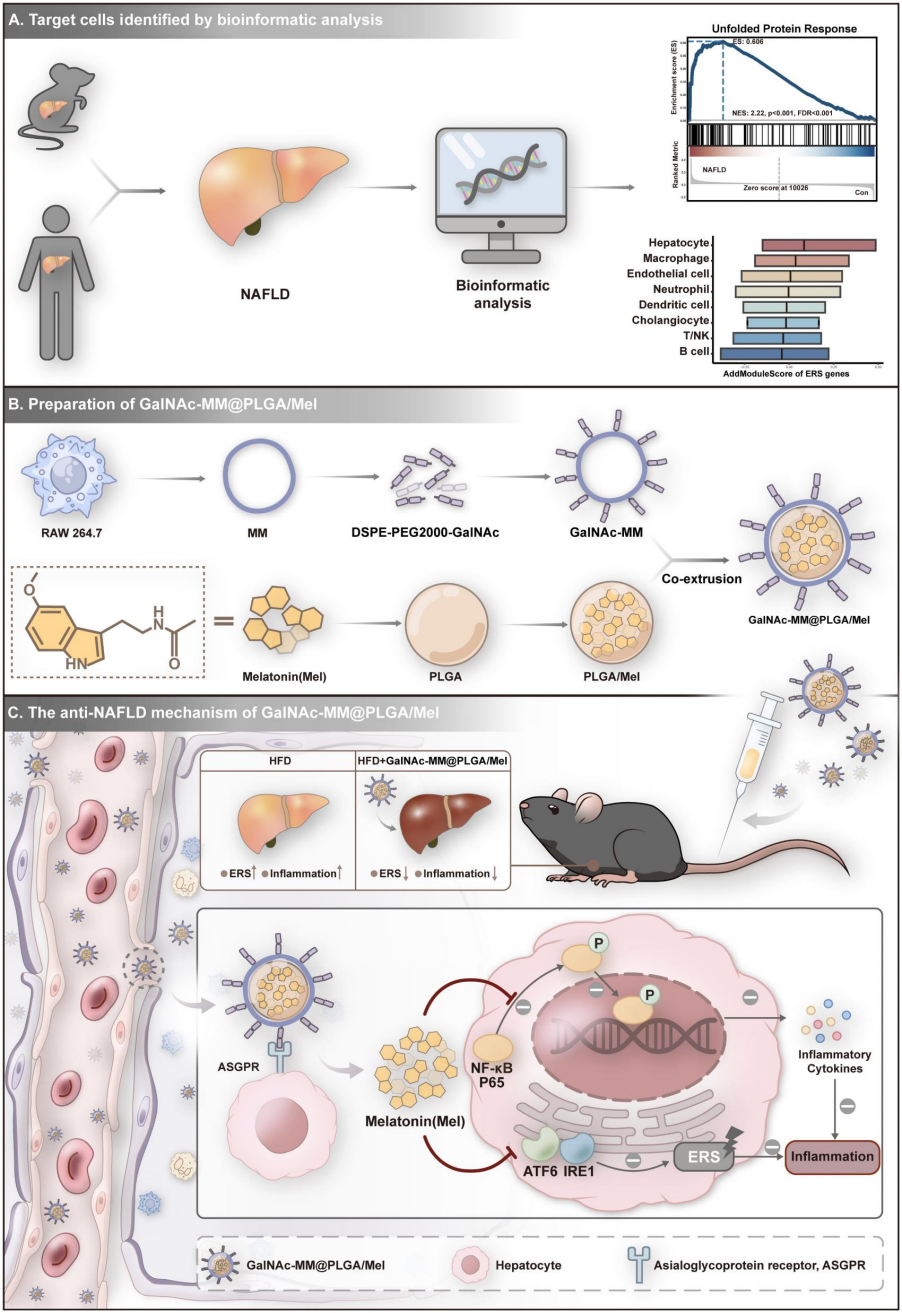
The schematic depicts the pathway of GalNAc-MM@PLGA/Mel in treating NAFLD. This approach presents numerous notable benefits: (**1**) The modification with GalNAc on the macrophage membrane enhanced the ability to target hepatocytes effectively. (**2**) The melatonin delivered by the NPs inhibited ERS and its downstream NF-κB signaling pathway, demonstrating significant anti-inflammatory and lipid-lowering effects.

In this research, we demonstrated that ERS is essential to the inflammatory response associated with NAFLD, as confirmed by means of bioinformatics analysis. Through examining single-cell sequencing data from liver tissues of NAFLD mice, we identified hepatocytes as the main cell type where ERS occurs. Building on these findings, we developed a NP coated with engineered macrophage membranes, referred to as GalNAc-MM@PLGA/Mel, to specifically target hepatocytes and alleviate ERS. The macrophage membrane provided the NP with bio-compatibility and improved cycling stability. Additionally, the modification with GalNAc on the macrophage membrane enhanced the NPs’ ability to target hepatocytes effectively. The Mel delivered by the NPs inhibited ERS and its downstream NF-κB signaling pathway, demonstrating significant anti-inflammatory and lipid-lowering effects. GalNAc-MM@PLGA/Mel NPs, in addition to maintaining the distinctive features of macrophage membrane-coated NPs, such as immune evasion, also exhibited specific targeting for hepatocytes. Benefiting from the targeted delivery design of the nanomaterial, Mel was able to exert therapeutic effects at a relatively low dose, thereby avoiding the potential risks of adverse effects associated with the higher systemic doses commonly used in previous studies. Therefore, this therapy, utilizing engineered cellular NPs, offers a safe, efficient and economically viable approach to treating NAFLD (Figure 1).

## Materials and methods

### Cell membrane extraction

The procedure for isolating cell membranes from RAW 264.7 cells followed an earlier established method [29]. In summary, cells were added to a hypotonic lysis solution composed of Tris-HCl (30 mM), d-mannitol (225 mM), sucrose (75 mM) and EGTA (0.2 mM), supplemented with protease inhibitors and phosphatase blocking agents. The suspension was then homogenized with a homogenizer. The resulting cell homogenate was subjected to centrifugation (10 000*g*, 4°C, 25 min), and the supernatant was harvested. The resulting supernatant underwent a second centrifugation (150 000*g*, 4°C, 30 min) to obtain the membrane pellet. The pellet was resuspended in an EDTA solution (0.2 mM) and subjected to another centrifugation (150 000*g*, 4°C, 30 min). Finally, the membrane pellet was resuspended in EDTA (0.2 mM), and the protein concentration was quantified using the BCA assay kit. The membrane suspension was then stored at −80°C for future use.

### Synthesis of cell engineering ligands

The synthesis of DSPE-PEG2000-GalNAc was performed based on previous literature [30]. A total of 20 mg of GalNAc was dissolved in 2 mL of pyridine. Then, succinic anhydride (1.0 equivalent [eq.]) and 4-dimethylaminopyridine (DMAP, 0.5 eq.) were added. The reaction took place for 6 h at 40°C. Afterward, the reaction solution was distilled and concentrated under reduced pressure. The product was then precipitated using a large volume of ice-cold ether and dried in a vacuum to obtain GalNAc-COOH. Next, weighed 100 mg of DSPE-PEG2000-NH2 and dissolved it in 3 mL of N,N-dimethylformamide (DMF). Next, added GalNAc-COOH (3.0 eq.), PyBOP (3.0 eq.) and triethylamine (5.0 eq.) to the reaction mixture. Allowed the reaction to rest at ambient temperature for 30 min. Following the reaction, concentrated the solution by distillation under reduced pressure, then dissolved the residue in ethanol. Transferred this solution to a dialysis bag (500 Da molecular weight cutoff [MWCO], Millipore). Dialysed the solution in pure water for 24 h. After dialysis, collected the dialysate and freeze-dried it to obtain DSPE-PEG2000-GalNAc. The ^1^H nuclear magnetic resonance spectra were measured using a Bruker Ascend 400 spectrometer.

### Preparation of biomimetic NPs

The preparation of PLGA/Mel NPs and membrane-coated PLGA/Mel NPs was conducted following a previously established protocol [29]. Briefly, we prepared a dichloromethane solution containing 50 mg PLGA and 2 mg Mel. This solution was then mixed with a 5% w/v polyvinyl alcohol (PVA) solution (RHAWN) and subjected to ultrasonication at 42 kHz and 100 W for 5 min while placed in a chilled bath, forming an emulsion. The prepared emulsion was subsequently mixed with 4 mL of a 1% w/v PVA solution and mixed under ambient conditions until complete evaporation of the methylene chloride. To eliminate residual free Mel and solvent residues, dialysis was performed in ultrapure water for 12 h utilizing a dialysis bag with a 3.5 kDa MWCO (Millipore). PLGA/Mel NPs were subsequently harvested by centrifugation (15 000*g*, 15 min) followed by lyophilization to yield the purified NPs. To prepare GalNAc-modified macrophage membranes, DSPE-PEG2000-GalNAc was conjugated to macrophage membrane vesicles using the lipid insertion method for 1 hour at 37°C. This was followed by sonication with a 100 W ultrasonic cell breaker for 2 s. To create membrane-coated PLGA/Mel NPs, macrophage membranes were mixed with PLGA NPs in a 1:1 ratio of membrane protein to PLGA by weight. This mixture underwent ultrasonication at 42 kHz and 100 W for 2 min using a bath sonicator. Finally, the mixture was extruded 15 times using a 200 nm polycarbonate porous membrane with a microextruder. After extruding to create a solution of membrane-coated PLGA/Mel NPs, the concentration was adjusted to 10 mg/mL by centrifuging (4500*g*, 15 min) using an Amicon ultracentrifugation device (30 kDa MWCO). The NPs were then harvested by further centrifuging at 15 000*g* for another 15 min and freeze-dried to yield purified NPs. GalNAc-MM@PLGA/Mel NPs were synthesized using the identical method, substituting GalNAc-modified membranes for the regular macrophage membranes. For the synthesis of MM@PLGA and PLGA NPs, the process was identical, but excluding the incorporation of Mel. MM@PLGA/DiI and PLGA/DiI NPs were produced similarly, substituting DiI for Mel, at a dye concentration of 0.1 wt% relative to the PLGA polymer. Likewise, MM@PLGA/DiR and PLGA/DiR NPs were synthesized using the identical method, with DiR replacing Mel, also at a concentration of 0.1% by weight of the PLGA polymer.

### Animal model of NAFLD

To model high-fat diet (HFD)-induced NAFLD, C57BL/6 mice were fed a high-fat diet consisting of 60% calories (D12492, Research Diets) from day −102 to day −1. On days 0, 7 and 14, the HFD-fed mice were treated with Mel, PLGA/Mel, MM@PLGA/Mel and GalNAc-MM@PLGA/Mel (1 mg/kg Mel each group). Mice treated with saline alone were used as the control group. On day 17, serum, liver, spleen, heart, lungs and kidneys were collected from all mice after euthanasia. All animal experiments were approved by the Institutional Animal Ethics Committee of the Second Affiliated Hospital of Chongqing Medical University (No. 2025-1029). The experimental procedures involving human liver samples in this study were approved by the ethics committee of the Second Affiliated Hospital of Chongqing Medical University (No. 2024-577). All liver specimens were flash frozen in liquid nitrogen, and further experiments were conducted in strict accordance with the Helsinki Declaration.

### Antibodies

The antibodies employed in this research are listed in detail in Supplementary Table S1.

Additional details are available in the Supplementary Materials.

## Results and discussion

### Mel-modulated ERS in hepatocytes to alleviate NAFLD

**Figure 2:**
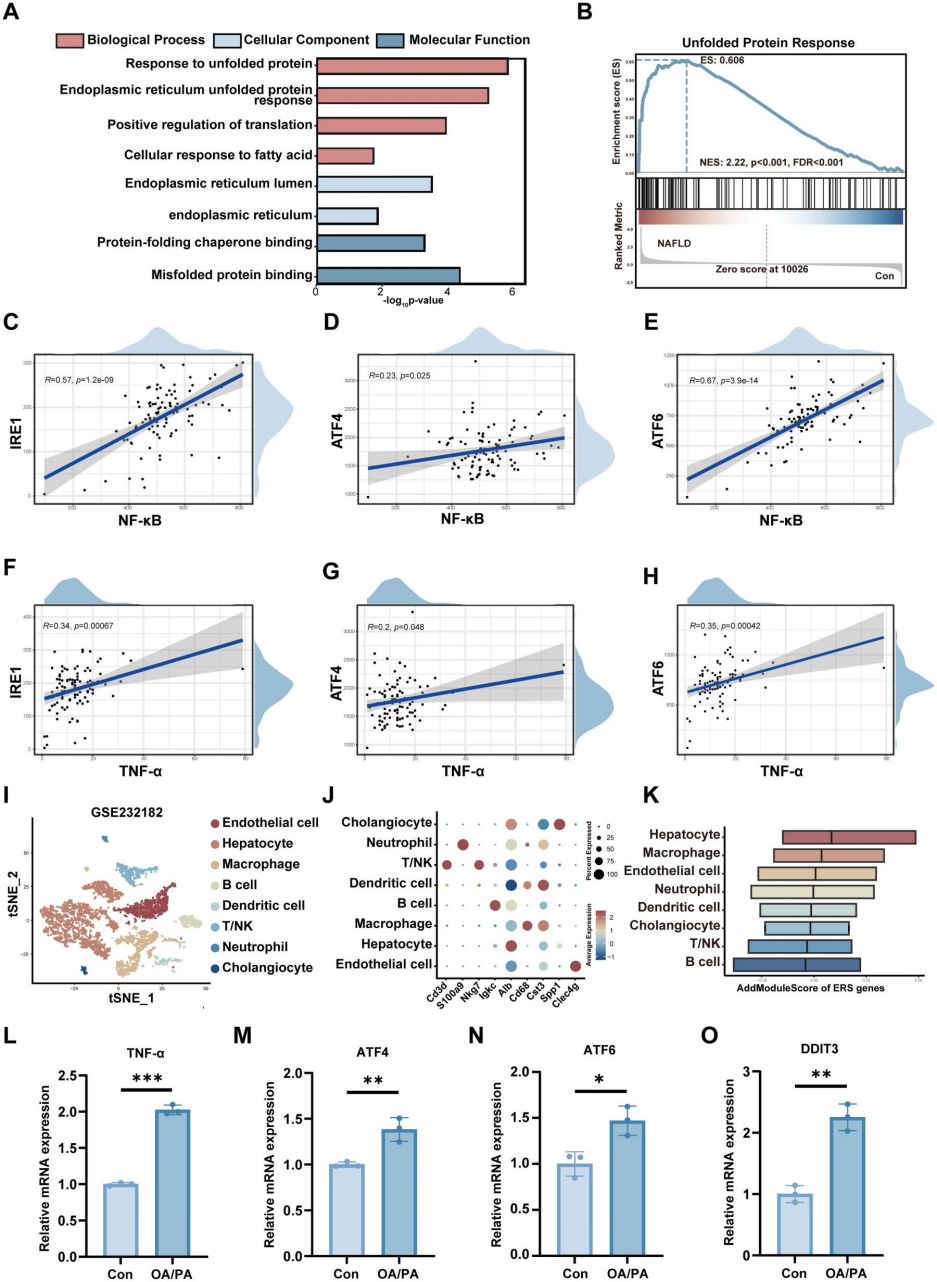
The role of ERS in the progression of NAFLD. (**A**) Biological processes of GO analysis for DEGs in hepatocytes under high-fat conditions compared with normal conditions, based on data from the GSE122660 dataset. (**B**) Biological processes of GSEA for DEGs in hepatocytes under high-fat conditions compared with normal conditions, based on data from the GSE122660 dataset. (**C–H**) Results of correlation analysis between ERS-related genes and inflammation-related genes in NAFLD liver tissues. Correlation coefficients *R* > 0 indicated positive correlation, *R* < 0 indicated negative correlation and *P* < 0.05 indicated a significant difference, data from the GSE167523 dataset. (**I**) t-SNE plot displaying the identified cell types using marker genes, based on data from the GSE232182 dataset. (**J**) Bubble chart illustrating the marker genes present in each cell cluster. (**K**) Box plots indicate the AddModuleScore of ERS genes for eight cell types in NAFLD. (**L–O**) PCR analysis of ERS-associated and inflammation-associated genes in AML12 cells prior to and following OA/PA treatment (*P < 0.05, **P < 0.01, ***P < 0.001).

To investigate the association between ERS pathway and NAFLD, we first conducted a GO (gene ontology) enrichment analysis on the GSE122660 dataset. The findings revealed that hepatocytes under high-fat conditions exhibited significantly enhanced ERS, accompanied by marked activation of the unfolded protein response (Figure 2A). Additionally, the Gene Set Enrichment Analysis (GSEA) revealed that this unfolded protein response was more prominently activated in hepatocytes exposed to high-fat conditions (Figure 2B). Importantly, many proteins present in NAFLD liver tissue undergo initial modifications and folding processes within the endoplasmic reticulum. To further explore the relationship between ERS and NAFLD, we focused on inflammatory genes associated with NAFLD: nuclear factor kappa-B (NF-κB) and tumor necrosis factor-α (TNF-α), analysing their correlation with genes indicative of ERS (GSE167523). As presented in Figure 2C–H, a notable positive relationship was found between the levels of NF-κB and IRE1 (immunoglobulin-regulated enhancer 1) in liver samples from NAFLD (*R* = 0.57, *P* = 1.2e-9). Additionally, a strong positive correlation was identified between NF-κB and ATF4 (activating transcription factor 4) (*R* = 0.23, *P* = 0.025), along with NF-κB and ATF6 (*R* = 0.67, *P* = 3.9e−14). Furthermore, TNF-α also exhibited strong positive associations with the expression of IRE1, ATF4 and ATF6 (IRE1: *R* = 0.34, *P* = 0.00067; ATF4: *R* = 0.20, *P* = 0.048; ATF6: *R* = 0.35, *P* = 0.00042). Collectively, our findings suggest a strong link between ERS-related genes and the development of NAFLD. This connection likely arises from the central role these genes play in the progression of NAFLD, induced by ERS, as well as their subsequent involvement in the unfolded protein response. To identify the primary cell types involved in ERS activation during NAFLD, we analysed single-cell datasets from the NAFLD model (Figure 2I and J). We calculated the average expression of the target gene set across different cell populations using the AddModuleScore function, which scores gene sets in single-cell RNA sequencing data. Our findings revealed that ERS-related genes showed higher expression in hepatocytes, as determined by the AddModuleScore function, suggesting a strong link between ERS and hepatocytes in NAFLD (Figure 2K).

**Figure 3:**
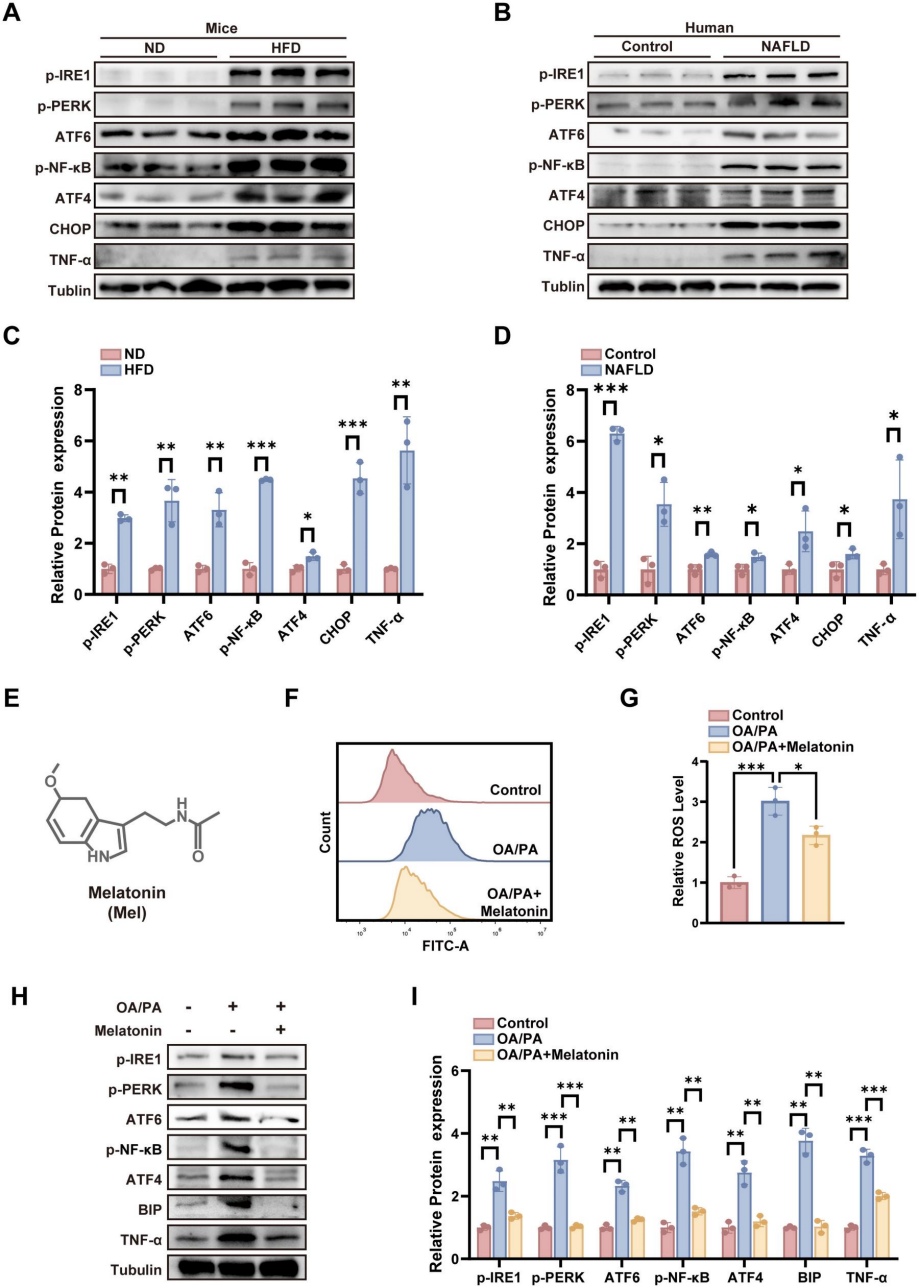
Effects of Mel on ERS modulation and its attenuation of NAFLD inflammatory response. (**A**) The protein levels of ERS-associated and inflammatory-associated molecules in hepatic tissue from control mice and NAFLD mice were determined by Western blotting (ND: normal diet, HFD: high-fat diet). (**B**) The protein levels of ERS-associated and inflammatory-associated molecules in liver tissue samples from normal human and those from NAFLD patients were determined by Western blotting. (**C**) Quantitative analysis of the data in mice. (**D**) Quantitative analysis of the data in humans. (**E**) Three-dimensional structure of melatonin. (**F**) Flow analysis of the expression level of reactive oxygen species in AML-12 cells after different treatments. (**G**) Quantitative analysis of the flow analysis data. (**H**) The protein levels of ERS-associated and inflammatory-associated molecules in different cell treatment groups were determined by Western blotting. (**I**) Quantitative analysis of the Western blotting data (*n* = 3, *P < 0.05, **P < 0.01, ***P < 0.001).

To validate the accuracy of the bioinformatics analysis results, we conducted a further examination of the gene expression profiles of both inflammatory and ERS-related genes at both cellular and tissue scales in NAFLD. At the cellular scale, an *in vitro* high-fat environment was simulated by stimulating AML12 cells with OA/PA (OA: oleic acid, PA: palmitic acid), providing a model to assess the effects of lipid-induced stress. RT-qPCR results indicated a notable upregulation of TNF-α expression in the OA/PA-treated group (Figure 2L). In parallel, the abundance of key molecules associated with ERS, such as ATF4, ATF6 and DDIT3 (DNA damage-inducible transcript 3), was substantially increased (Figure 2M–O). At the histological level, liver samples from HFD-induced NAFLD model mice and NAFLD patients were analysed using Western blot analysis. Results showed significantly increased expression levels of several proteins associated with the ERS in HFD mice, including phosphorylated IRE1 (p-IRE1), phosphorylated PERK (p-PERK), ATF6, ATF4, CHOP (encoded by DDIT3), as well as the inflammatory markers NF-κB and TNF-α (Figure 3A and C). Similarly, liver samples from NAFLD patients exhibited significantly higher expression of ERS-associated proteins (p-IRE1, p-PERK, ATF6, ATF4 and CHOP) and inflammation-related proteins (NF-κB and TNF-α) compared with the healthy controls (Figure 3B and D). Considering these findings, we infer that ERS pathway is crucial for the progression of NAFLD.

Mel (Figure 3E) is a hormone secreted by the pineal gland that has significant antioxidant and anti-inflammatory properties. It also acts as a novel selective inhibitor of ATF6, leading us to hypothesize that Mel may have therapeutic effects on NAFLD through the modulation of ERS pathway. To investigate its specific mechanism of action, we treated AML12 cells with Mel after OA/PA intervention. Using flow cytometry, we evaluated changes in intracellular ROS levels. The results showed that OA/PA exposure markedly increased intracellular ROS accumulation in AML12 cells, whereas Mel treatment significantly attenuated this increase (Figure 3F and G). As shown in Figure 3H and I, treatment with OA/PA led to a significant upregulation of p-NF-κB and TNF-α protein levels in AML12 cells, but these increases were markedly reduced by Mel treatment. Under normal conditions, the abundance of ERS-associated molecules—such as p-IRE1, p-PERK, ATF6, ATF4 and BIP—was minimal in AML12 cells. However, OA/PA stimulation significantly increased the expression of these proteins, and Mel was able to mitigate this increase by suppressing the expression of ERS-associated proteins. To examine the involvement of ERS in the therapeutic impact of Mel on NAFLD, we employed the ERS antagonist 4-PBA (4-phenylbutyric acid, Supplementary Figure S1A). Our results showed that 4-PBA markedly decreased the levels of ERS-associated molecules (including ATF4, ATF6 and DDIT3) in AML12 and suppressed inflammatory response, demonstrated through a notable reduction in TNF-α expression in comparison with the OA/PA group (Supplementary Figure S1B–E). Upon co-administration of Mel with 4-PBA, a further decrease in the expression of ATF4, ATF6 and DDIT3 was detected. In parallel, inflammatory responses in AML12 cells were also diminished, as indicated by reduced TNF-α expression. These findings suggest that Mel effectively suppresses inflammation induced by a high-fat environment in hepatocytes, likely by modulating intracellular ERS.

In summary, our bioinformatics analysis indicates that ERS in hepatocytes is essential for the inflammatory response linked to NAFLD in both human patients and animal models. ERS is particularly prevalent in NAFLD and plays a crucial role in the progression of this disease [3]. At present, only a few drugs have shown positive therapeutic effects in NAFLD by inhibiting ERS. For example, one study demonstrated that metformin may prevent NAFLD by inhibiting the IRE1/XBP1 signaling pathway, promoting β-cell proliferation and improving insulin resistance [31]. In addition, empagliflozin treatment has been shown to markedly ameliorate hepatic histopathological injury in high-fat diet-fed mice, reduce lipogenic and inflammatory markers, downregulate ERS-related molecules and concurrently enhance autophagy while attenuating apoptosis [32]. Likewise, umbelliferone treatment, in both high-fat diet-fed mice and OA/PA-treated AML12 cells, was found to reduce lipid droplet accumulation, decrease hepatic triglyceride levels and insulin resistance and suppress ERS as well as hepatocellular apoptosis [33]. Nevertheless, although interventions targeting ERS have shown promising effects in experimental models of NAFLD, this field remains largely at the preclinical stage [34].

Mel, an indoleamine with antioxidant, anti-inflammatory and metabolic regulatory properties [9], highlights its potential therapeutic value for the treatment of NAFLD. For example, studies have shown that Mel can improve glucose tolerance and insulin sensitivity in high-fat diet-induced mice, while reducing hepatic lipid accumulation, thereby partially correcting hepatic metabolic disturbance [35]. In addition, in ob/ob mouse models, Mel treatment has been reported to markedly ameliorate hepatic histopathological and ultrastructural damage, attenuate the degree of steatosis and partially restore organelle dysfunction, demonstrating a direct protective effect against hepatocellular injury [36]. Mel has also been shown to alleviate high-fat diet-induced hepatic metabolic abnormalities and steatosis by regulating the microRNA-34a-5p/SIRT1 axis, enhancing autophagic activity and improving mitochondrial morphology and function [37]. Notably, in high-fat diet-induced NASH models, Mel has been demonstrated to suppress the activation of inflammasome-related signaling, thereby reducing hepatic inflammatory responses and, to some extent, ameliorating fibrosis-related changes [38]. More recent studies have further suggested that Mel can attenuate oxidative damage and ferroptosis, thereby improving NAFLD-associated inflammatory responses and fibrotic alterations [39]. These findings are consistent with our results, our *in vitro* cellular model, which simulates high-fat conditions via OA/PA treatment, demonstrates that Mel effectively suppresses hepatocyte activation by modulating ERS. This action ultimately reduces inflammatory cytokine secretion and alleviates inflammatory responses.

Based on these results, we propose a nano-therapeutic approach that targets hepatocyte ERS to treat NAFLD.

### Preparation and characterization of GalNAc-MM@PLGA/Mel

**Figure 4:**
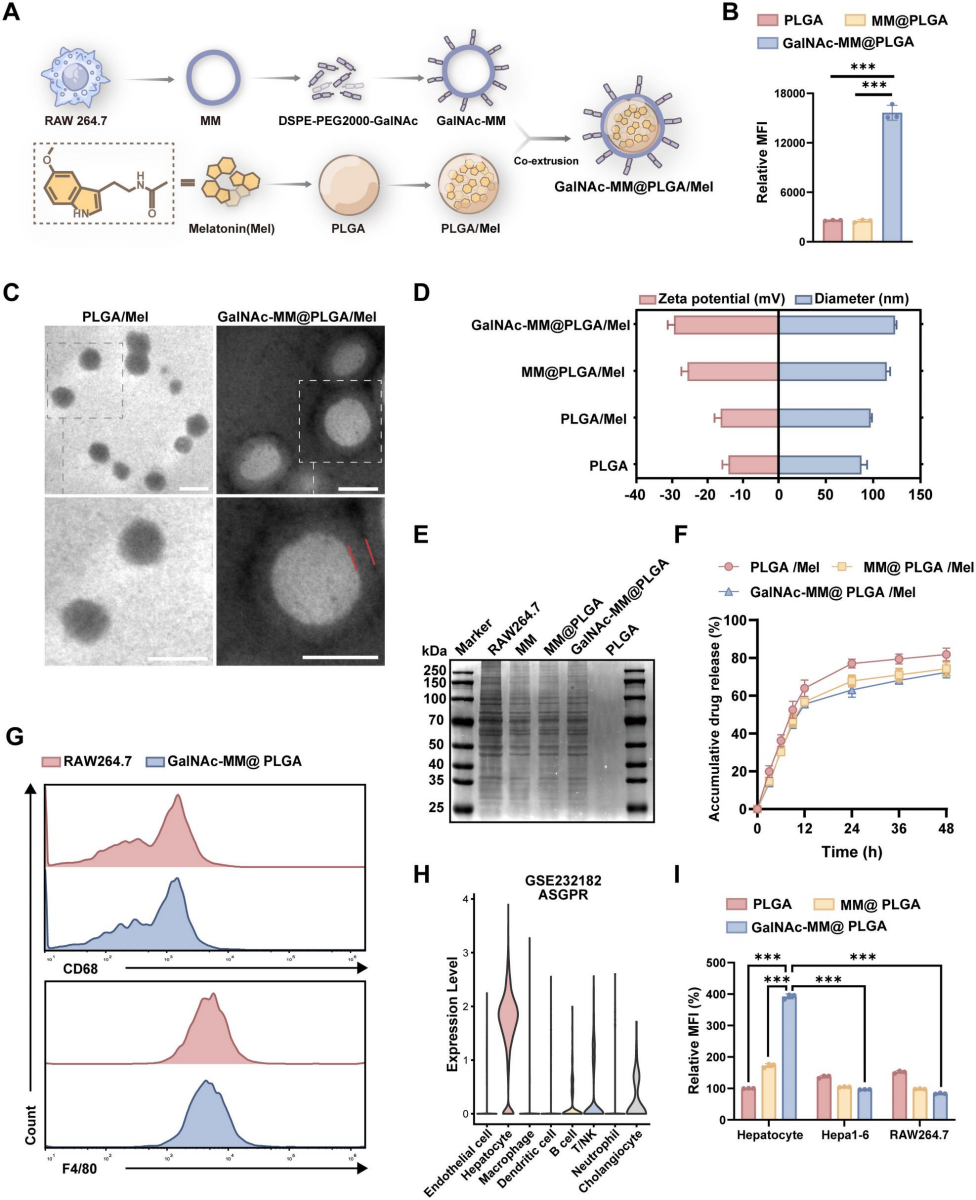
Characterization and synthesis of GalNAc-MM@PLGA/Mel. (**A**) Illustrative representation of the fabrication procedure for GalNAc-MM@PLGA/Mel NPs. (**B**) Determination of fluorescence intensity of HepG2 cells cultured with different DiI-labeled NPs. (**C**) Typical TEM images of PLGA/Mel and GalNAc-MM@PLGA/Mel NPs (Scale bar, 100 nm). (**D**) Measurement of mean particle size and *ζ*-potential for NPs. (**E**) SDS-PAGE results of protein in each group. (**F**) *In vitro* Mel release curves for PLGA/Mel, MM@PLGA/Mel and GalNAc-MM@PLGA/Mel NPs. (**G**) Flow cytometry evaluation of macrophage markers CD68 and F4/80 in RAW264.7 cells and GalNAc-MM@PLGA NPs. (**H**) Distribution and quantitative assessment of ASGPR expression across all cell types using the GSE232182 single-cell dataset. (**I**) Determination of fluorescence intensity of primary mouse hepatocytes, Hepa1-6 and RAW264.7 cells cultured with different DiI-labeled NPs (*n* = 3, ***P < 0.001).

Based on the results of our bioinformatics analysis, we developed a novel NP dedicated to effectively inhibiting hepatocyte ERS pathway and significantly alleviating the symptoms of NAFLD, which provides a new approach to NAFLD treatment. Because the desialylated glycoprotein receptor (ASGPR) is abundantly and specifically expressed on hepatocytes, its ligand, N-acetylgalactosamine (GalNAc), serves as an effective targeting moiety for designing nanodelivery systems with improved specificity and efficiency toward hepatocytes [40]. The synthesis process of GalNAc-MM@PLGA/Mel NPs is illustrated in Figure 4A and Supplementary Figure S2A and B. We further investigated whether GalNAc influenced the targeting effectiveness of GalNAc-MM@PLGA. Human hepatocarcinoma (HepG2) cells, which have a high expression of ASGPR [41], were selected for phagocytosis experiments. For these experiments, we utilized DiI-labeled PLGA NPs. After co-incubating these NPs with HepG2 cells, we measured the cells’ fluorescence intensity by flow cytometry to evaluate the cellular uptake of GalNAc-MM@PLGA/DiI, MM@PLGA/DiI and PLGA/DiI. Our findings showed that the cellular intake of GalNAc-MM@PLGA was substantially higher than that of either PLGA or MM@PLGA (Figure 4B). Additionally, we successfully synthesized PLGA NPs containing Mel (PLGA/Mel) using a nanoprecipitation method. Characterization by UV/Vis spectroscopy indicated that these NPs contained 2.56% ± 0.12% of Mel and achieved an encapsulation efficiency of 78.43% ± 0.31%. Subsequently, we generated PLGA/Mel NPs coated with GalNAc-modified macrophage membranes (GalNAc-MM) through the co-extrusion process. Transmission electron microscopy (TEM) revealed that the PLGA/Mel NPs displayed a spherical structure and that the GalNAc-MM@PLGA/Mel NPs showed a distinct core–shell architecture (Figure 4C). Dynamic light scattering (DLS) technique results showed that the mean particle diameter of the GalNAc-MM@PLGA/Mel NPs was about 122.9 ± 2.1 nm, with a *ζ*-potential of −29.4 ± 3.2 mV and a polydispersity index (PDI) of 0.191 ± 0.035 (Figure 4D). Notably, when GalNAc-MM@PLGA/Mel NPs were incubated in DMEM medium, no appreciable changes in particle dimensions were detected at room temperature, suggesting that the colloidal suspension remained stable (Supplementary Figure S3). Additionally, Kaumas Brilliant Blue staining revealed the presence of membrane proteins on the surface of GalNAc-MM@PLGA (Figure 4E). Together, these findings offer strong evidence for the successful synthesis of NPs coated by GalNAc-modified macrophage membranes.

We evaluated the drug release kinetics of three types of NPs—GalNAc-MM@PLGA/Mel, MM@PLGA/Mel and PLGA/Mel—in PBS buffer (37 °C, pH 7.4). After incubating for 48 h, the release rates of Mel were determined as 81.8% for PLGA/Mel, 74.1% for MM@PLGA/Mel and 72.3% for GalNAc-MM@PLGA/Mel. While the Mel release profiles of both MM@PLGA/Mel and GalNAc-MM@PLGA/Mel were moderately lower than those of PLGA/Mel, no substantial difference was detected between the two NPs (Figure 4F). Flow cytometry analysis revealed that GalNAc modification did not significantly alter the expression of macrophage-specific biomarkers, CD68 and F4/80 (Figure 4G). This suggests that the physical and functional stability of the macrophage membrane, which is essential to preserving the physiological activity of membrane-coated NPs, was effectively maintained throughout the formulation of GalNAc-MM@PLGA/Mel NPs. Thereafter, we evaluated the impact of these NPs on cell viability via the Cell Counting Kit-8 (CCK-8) assay. As illustrated in Supplementary Figure S4, co-incubation of AML12 cells with PLGA, MM@PLGA and GalNAc-MM@PLGA NPs for 24 h at doses of 10, 50, 100 and 200 μg/mL did not lead to a notable difference in cell viability. These results showed that PLGA, MM@PLGA and GalNAc-MM@PLGA NPs exhibit good cytocompatibility.

Overall, these findings confirm the successful fabrication of GalNAc-MM@PLGA/Mel and its functional integrity, which are essential for ensuring effective biological activity. Additionally, the findings provide comprehensive support for the biosafety of these NPs.

### *In vitro* evaluation of the immune-evasive properties and hepatocyte-targeting capacity of GalNAc-MM@PLGA NPs

Currently, many drug delivery platforms are eliminated by the mononuclear phagocyte system before arriving at the destination site [42, 43]. However, there is growing evidence that macrophage membrane-coated NPs can evade immune phagocytosis *in vivo* [44, 45]. Accordingly, we used RAW 264.7 cells to examine the uptake efficiency of PLGA, MM@PLGA and GalNAc-MM@PLGA NPs. We labeled PLGA with DiI and measured average cellular fluorescence intensity by flow cytometry. As illustrated in Supplementary Figure S5A–C, macrophages exposed to PLGA/DiI for 4 h displayed strong fluorescence signals, indicating substantial intake of PLGA NPs by RAW 264.7 cells. Conversely, weaker fluorescence intensity was observed in macrophages treated with MM@PLGA/DiI or GalNAc-MM@PLGA/DiI. Moreover, the GalNAc modification did not notably affect the uptake of GalNAc-MM@PLGA/DiI by macrophages, indicating that GalNAc modification does not further boost the immune-evasive properties of these NPs.

Next, we assessed the *in vitro* targeting ability of GalNAc-MM@PLGA NPs. Because ASGPR expression on the cell surface is crucial for effective NP targeting, we analysed the GSE232182 single-cell dataset to determine which cell populations predominantly express this receptor. The results showed that ASGPR expression was enriched in hepatocytes, identifying them as the principal ASGPR-positive cell type in the liver (Figure 4H). To validate this finding *in vitro*, we isolated primary mouse hepatocytes using collagenase perfusion. After that, we co-incubated PLGA/DiI, MM@PLGA/DiI and GalNAc-MM@PLGA/DiI with primary mouse hepatocytes, Hepa1-6 (mouse hepatocarcinoma cells) and RAW264.7 (mouse mononuclear macrophage cells). Subsequent flow cytometric analysis was performed to quantify the mean fluorescence intensity in each cell type. As illustrated in Figure 4I, the cellular intake of GalNAc-MM@PLGA in primary mouse hepatocytes was significantly higher than that of PLGA or MM@PLGA. In contrast, the addition of GalNAc did not significantly enhance the cellular uptake of NPs in Hepa1.6 and RAW264.7 cells. Furthermore, GalNAc-MM@PLGA demonstrated significantly higher uptake in primary mouse hepatocytes compared to Hepa1.6 or RAW264.7 cells, which further substantiates its hepatocyte-specific targeting properties *in vitro*. Subsequently, we explored the effects of PLGA/Mel NPs *in vitro*. Using flow cytometry, we evaluated changes in intracellular ROS levels after PLGA/Mel NPs treatment. As shown in Supplementary Figure S6A and B, the level of ROS was significantly enhanced by OA/PA stimulation, while the PLGA/Mel NPs significantly inhibited intracellular ROS accumulation. In contrast, PLGA NPs did not affect the ROS level in the cells. We explored the *in vitro* effects of PLGA/Mel NPs on ERS pathway and inflammation pathway activity in AML-12 cells. As shown in Supplementary Figure S6C and D, immunoblotting assays revealed that the incorporation of PLGA/Mel NPs significantly inhibited the expression of ERS-associated proteins and inflammation-associated proteins in AML-12 cells. In contrast, PLGA NPs did not affect ERS or inflammation pathway activity in the cells.

In conclusion, our findings indicate that PLGA NPs coated with macrophage membranes effectively evade disposal by the macrophages. This phenomenon has been validated and observed in numerous studies [44, 45]. Nevertheless, the modification of GalNAc on the cell membrane did not significantly enhance this effect. Our study further demonstrated that GalNAc-MM@PLGA NPs notably improved targeting to hepatocytes *in vitro*. These findings highlight the promise of GalNAc-modified NPs for achieving both reduced immune clearance and improved cell-specific drug delivery.

### *In vivo* targeting and therapeutic effects of GalNAc-MM@PLGA NPs

**Figure 5:**
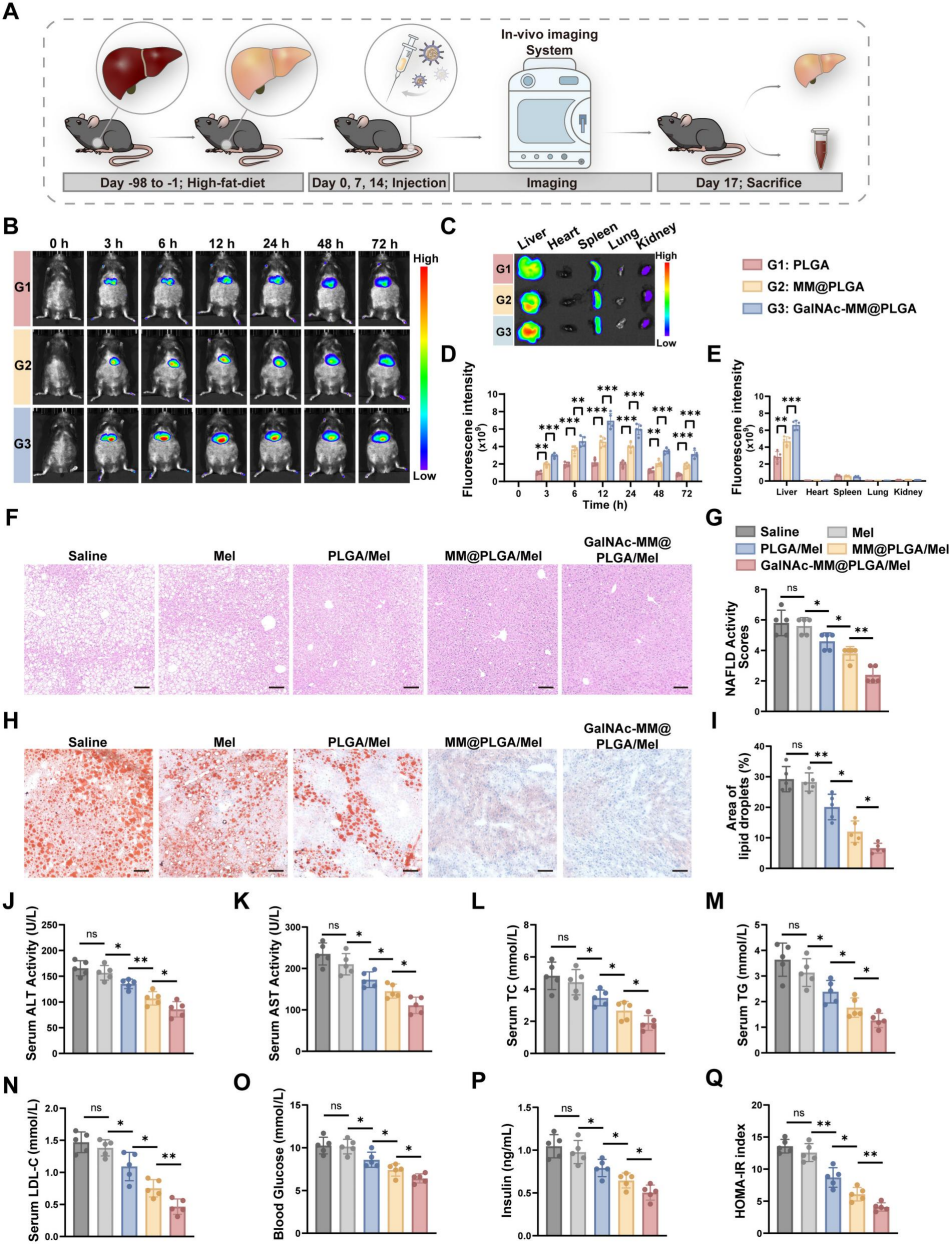
Coating with macrophage membranes improves the targeting efficacy of NPs toward hepatocytes and alleviates NAFLD. (**A**) Illustration of the *in vivo* therapeutic regimen. (**B**) *In vivo* fluorescence imaging at different time points after the dosing of different coated NPs in mice with NAFLD. PLGA group with administration of PLGA/DiR, MM@PLGA group with administration of MM@PLGA/DiR, GalNAc-MM@PLGA group with administration of GalNAc-MM@PLGA/DiR. (**C**) Fluorescence imaging of isolated organs (liver, heart, spleen, lung and kidney) from NAFLD mice after 72 h injection of different DiR-labeled NPs. (**D**) Quantitative assessment of NPs fluorescence signals in the livers of NAFLD mice at various time points. (**E**) Quantitative measurement of fluorescence in isolated organs. (**F**) Representative H&E images from NAFLD murine model administered with different treatments (scale bar, 100 μm). (**G**) Assessment of NAFLD activity scores across different treatment groups. (**H**) Representative Oil Red O staining liver images from NAFLD murine model administered with different treatments (scale bar, 100 μm). (**I**) Quantitative analysis of lipid droplets across the treatment groups. (**J and K**) ELISA data showing serum ALT and AST activities in different groups. (**L–N**) Metabolic parameters (triglyceride, low-density lipoprotein and cholesterol concentrations) from different treatment groups. (**O–Q**) Glucose metabolism parameters (glucose, insulin and HOMA-IR) from different treatment groups (*n* = 5, ‘ns’ denotes no significance, *P < 0.05, **P < 0.01, ***P < 0.001).

We further explored the *in vivo* targeting efficacy of GalNAc-MM@PLGA NPs in a NAFLD model (Figure 5A). The NAFLD model was established using a previously described method. To evaluate the targeting and distribution of the NPs *in vivo*, PLGA NPs were tagged with DiR and administered by tail vein injection. An *in vivo* imaging system was employed to observe the accumulation of PLGA/DiR, MM@PLGA/DiR and GalNAc-MM@PLGA/DiR in the liver at various time points (Figure 5B and D). These data indicated that hepatic drug accumulation began as early as 3 h post-administration, with DiR fluorescence persisting in hepatic tissue for over 72 h. Throughout the study, the PLGA/DiR group exhibited weak fluorescent signals in the liver, whereas the MM@PLGA/DiR group exhibited stronger fluorescence. Notably, GalNAc-MM@PLGA/DiR NPs exhibited superior targeting to inflammatory liver sites compared to MM@PLGA/DiR NPs. To further assess the biodistribution of these NPs across different organs, we collected liver and other major organs at 72 h post-injection for fluorescence intensity analysis (Figure 5C and E). The data indicated that the fluorescence signal in hepatic tissue was significantly higher with GalNAc-MM@PLGA/DiR NPs than with either MM@PLGA/DiR or PLGA/DiR NPs. This indicates that the presence of GalNAc on macrophage membranes enhances NP targeting to inflamed injury sites. Notably, GalNAc-MM@PLGA/DiR showed relatively lower fluorescence in other organs, while PLGA/DiR NPs exhibited fluorescence signals in the spleen and renal organ. Our results suggest that NPs lacking cell membrane coating are speedily eliminated by the immune system *in vivo*. Furthermore, fluorescence signals in other tissues, such as the spleen, were similar for both MM@PLGA/DiR and GalNAc-MM@PLGA/DiR, suggesting that GalNAc modification does not boost the immune evasion capability of cell membrane-coated NPs. This finding is consistent with our *in vitro* observations.

Following the evaluation of the targeting efficiency of GalNAc-MM@PLGA/Mel, we proceeded to evaluate the treatment efficacy of these NPs *in vivo*. We established a mouse model for NAFLD by means of prolonged feeding with a high-fat diet [46]. Thereafter, we administered saline, Mel, PLGA/Mel, MM@PLGA/Mel and GalNAc-MM@PLGA/Mel weekly via tail vein injection. To evaluate liver damage, we conducted hematoxylin and eosin (H&E) staining and NAFLD activity scoring. As shown in Figure 5F and G, the saline and Mel groups showed extensive liver tissue damage, which was characterized by prominent hepatic steatosis, lobular inflammation and ballooning degeneration of hepatocytes. In comparison, the PLGA/Mel group showed a mild improvement in liver tissue damage. The MM@PLGA/Mel group showed significantly reduced vacuolation and hepatocyte necrosis, while the GalNAc-MM@PLGA/Mel group experienced the least amount of liver injury. Additionally, Oil Red O staining revealed that both the saline and Mel groups exhibited considerable hepatic steatosis, although a slight improvement was observed in the PLGA/Mel group. Conversely, the MM@ PLGA/Mel group displayed a notable decline in the extent of hepatic steatosis. Furthermore, the GalNAc-MM@ PLGA/Mel treatment resulted in the most pronounced decrease in lipid droplet accumulation within the cytoplasm (Figure 5H and I). Biochemical assays further supported these findings. In comparison with the saline group, serum ALT and AST activities were slightly decreased in the Mel and PLGA/Mel groups. However, significant reductions in these enzymes were detected in the MM@PLGA/Mel and GalNAc-MM@PLGA/Mel groups, with the latter showing the greatest decline in both serum ALT and AST activities, thereby offering the strongest protective benefit (Figure 5J and K). Moreover, we investigated the effects of the GalNAc-MM@PLGA/Mel on metabolic indicators [47]. Mice treated with GalNAc-MM@PLGA/Mel showed significant decreases in serum and hepatic triglyceride (TG), total cholesterol (TC) and low-density lipoprotein levels compared to the other groups (Figure 5L–N and Supplementary Figure S7A and B). Subsequently, we investigated the potential effects of GalNAc-MM@PLGA/Mel on glucose metabolism [48]. Compared with the other groups, mice treated with GalNAc-MM@PLGA/Mel exhibited significantly lower blood glucose and insulin levels. In addition, the homeostasis model assessment of insulin resistance (HOMA-IR) index was markedly reduced, suggesting that GalNAc-MM@PLGA/Mel treatment alleviated insulin resistance in mice (Figure 5O–Q). These findings indicate that GalNAc-MM@PLGA/Mel offers the most potent therapeutic effect for NAFLD.

In conclusion, our experimental findings demonstrate that GalNAc-MM@PLGA NPs can effectively direct to inflammatory regions in NAFLD. This effectiveness is likely due to the macrophage membranes providing immune evasion for the PLGA NPs, coupled with the GalNAc modification, which enhances NPs accumulation at NAFLD sites by specifically targeting ASGPR on hepatocyte membranes. These enhancements improve the targeting efficiency of PLGA NPs, potentially suppressing inflammatory cascades in the initial phases of NAFLD. Additionally, we observed that GalNAc-MM@PLGA/Mel effectively reduced liver damage in a NAFLD mouse model. The NPs demonstrated strong targeting of liver injury sites, facilitating more efficient drug delivery and reducing damage. These results validate the targeted therapeutic efficacy of GalNAc-MM@PLGA/Mel in alleviating liver damage in the NAFLD murine model, confirming the NPs’ effectiveness *in vivo*.

### *In vivo* anti-ERS activity of GalNAc-MM@PLGA NPs

**Figure 6:**
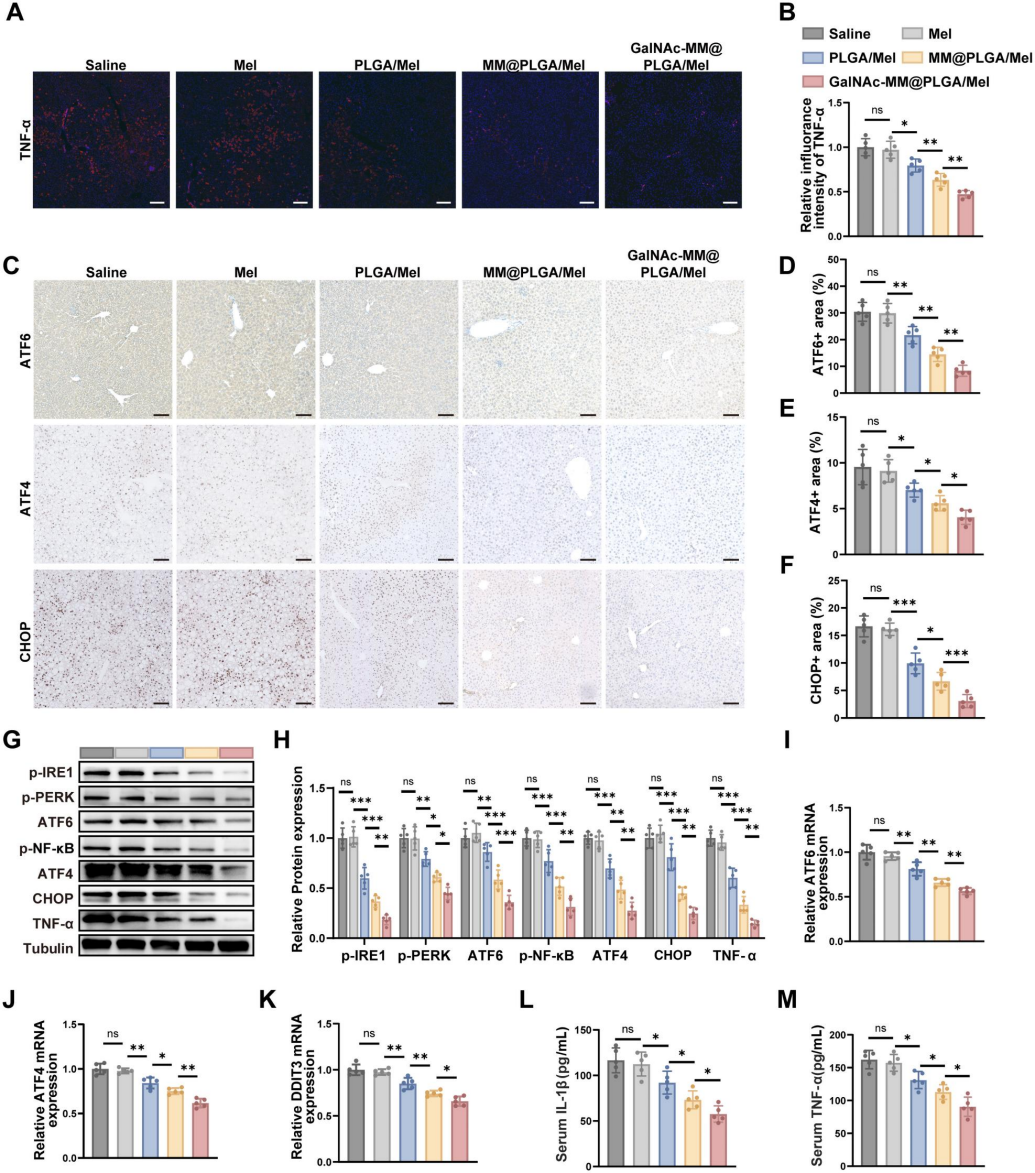
*In vivo* anti-ERS effect of GalNAc-MM@PLGA NPs. (**A**) Immunofluorescence staining of TNF-α in liver tissues from various NAFLD mice groups (scale bar, 200 μm). (**B**) Quantitative analysis of TNF-α based on immunofluorescence images. (**C**) Immunohistochemical staining for ATF4, ATF6 and CHOP in liver tissues from different NAFLD mice groups (scale bar, 100 μm). (**D–F**) Quantitative analysis of ATF4, ATF6 and CHOP from immunohistochemical images. (**G**) Representative images showing the expression of hepatic ERS-associated proteins and inflammatory-related proteins in different NAFLD murine groups. (**H**) Analysis of the Western blotting results. (**I–K**) PCR data of ERS-associated genes in different NAFLD mice groups. (**L and M**) Serum levels of inflammatory cytokines in different NAFLD groups, detected via ELISA. (*n* = 5, ‘ns’ indicates no significance, *P < 0.05, **P < 0.01, ***P < 0.001).

To investigate whether GalNAc-MM@PLGA/Mel could trigger an anti-inflammatory activity *in vivo*, we measured inflammatory factors TNF-α [49] in NAFLD using immunofluorescence staining. Strong TNF-α fluorescence was observed in the saline and Mel groups, while a moderate reduction was detected in the PLGA/Mel group. Conversely, treatment with MM@PLGA/Mel markedly diminished TNF-α signal intensity, and the lowest fluorescence levels were observed following GalNAc-MM@PLGA/Mel administration (Figure 6A and B). These findings indicate that GalNAc-MM@PLGA/Mel exerts a significant *in vivo* anti-inflammatory activity.

We then explored whether macrophage membrane-coated NPs could regulate the activation of the ERS pathway in NAFLD *in vivo*. Immunohistochemical experiments were performed to assess levels of key ERS-related molecules, including ATF6, ATF4 and CHOP. Compared with the saline- and Mel-treated groups, the PLGA/Mel treatment resulted in a modest reduction in ATF6 expression. Conversely, both MM@PLGA/Mel and GalNAc-MM@PLGA/Mel treatments produced a marked decrease in ATF6-positive regions, with a more pronounced effect in the GalNAc-MM@PLGA/Mel group. Similar results were obtained for ATF4 and CHOP assays. The saline-treated and Mel-treated groups exhibited high expression of ATF4 and CHOP, whereas the PLGA/Mel-treated group showed a slight reduction in the positive areas for these markers. However, significant decreases in ATF4 and CHOP expression were evident in the MM@PLGA/Mel group, with the GalNAc-MM@PLGA/Mel group showing the most pronounced decrease (Figure 6C–F). These findings suggest that GalNAc-MM@PLGA/Mel plays a significant role in inhibiting ERS activation in NAFLD model mice. These results were further validated by protein blotting data. Our findings indicated that TNF-α expression decreased slightly after PLGA/Mel treatment compared with the saline- and Mel-treated groups, with a more significant decrease observed after MM@PLGA/Mel treatment. The most substantial decreases in TNF-α protein levels were detected after treatment with GalNAc-MM@PLGA/Mel, indicating a substantial decrease in inflammatory markers. The expression of ATF6, ATF4 and CHOP followed a similar trend. These molecules were notably expressed in both the saline and Mel-treated groups, with a slight decrease in expression observed in the PLGA/Mel group. Conversely, the MM@PLGA/Mel treatment resulted in a marked decline in the levels of these molecules, with the most prominent decrease observed in the GalNAc-MM@PLGA/Mel group (Figure 6G and H). The observed outcomes align with our *in vitro* results, indicating that GalNAc-MM@PLGA/Mel NPs effectively suppress tissue inflammation and ERS *in vivo*. To further confirm our findings, we conducted qPCR to assess the expressions of ERS-associated molecules in murine hepatic tissue. The PLGA/Mel group exhibited a slight decrease in ATF6 expression levels. However, both the MM@PLGA/Mel and GalNAc-MM@PLGA/Mel groups displayed more substantial reductions, with the GalNAc-MM@PLGA/Mel group demonstrating the most evident decrease (Figure 6I). Similar results were noted for ATF4 and DDIT3, where the most substantial reductions in expression levels were also found in the GalNAc-MM@PLGA/Mel group (Figure 6J and K). Additionally, we used ELISA to quantitatively assess the levels of inflammatory mediators TNF-α and IL-1β in the serum of NAFLD mice. In the PLGA/Mel group, serum concentrations of TNF-α and IL-1β were moderately reduced. However, a more substantial decrease in these cytokine levels was detected in the MM@PLGA/Mel group. Remarkably, the GalNAc-MM@PLGA/Mel group treatment resulted in the most significant decrease in the levels of these pro-inflammatory cytokines compared to all other groups (Figure 6L and M).

In conclusion, our *in vivo* findings demonstrated that loading GalNAc-modified macrophage membranes reduces the inflammatory response in NAFLD while inhibiting the activation of the ERS pathway. This effect is due to the artificial assembly of GalNAc on the macrophage membranes, which enhances the efficient delivery of drug-loaded NPs by allowing them to specifically target hepatocytes at the site of inflammation. As a result, this process decreases the release of inflammatory factors and reduces the infiltration of inflammatory cells. Additionally, GalNAc-MM@PLGA/Mel NPs proved effective in alleviating liver injury caused by NAFLD and modulating the ERS pathway to reduce cellular stress. The presence of GalNAc improved the NPs’ ability to target inflammation sites specifically, resulting in a pronounced drop in the expression of ERS-related molecules and further diminishing the activation of the ERS pathway. Consequently, this action helps alleviate inflammatory responses and liver tissue damage. These results underscore the potential of GalNAc-modified, cell membrane-coated NPs to mitigate the ERS pathway and inflammation. Overall, our study suggests that GalNAc-MM@PLGA/Mel is a promising therapeutic tool for NAFLD, effectively inhibiting intracellular ERS pathway activation and suppressing localized inflammation *in vivo*. Notably, the therapeutic efficacy of the present strategy was evaluated only in a male NAFLD mouse model. Male mice were preferentially used for two main reasons. First, in diet-induced NAFLD/MASLD models, males generally develop more stable hepatic lipid accumulation, insulin resistance and more pronounced liver pathological changes, and are therefore widely used in preclinical studies [50]. Second, both clinical and experimental evidence have demonstrated clear sex differences in the onset and progression of NAFLD, with females of reproductive age generally showing greater protection than males. This phenomenon is widely considered to be closely associated with the hepatoprotective effects of estrogen signaling [51]. Nevertheless, although the present study was conducted only in male mice, the key pathological basis and targeting rationale of this nano-therapeutic strategy—including hepatocellular ERS, oxidative stress and the hepatocyte target ASGPR—are not male-specific. This suggests that the therapeutic strategy may also have potential applicability in females. However, this possibility still needs to be further validated in female NAFLD models.

### Biosafety of GalNAc-MM@PLGA NPs

**Figure 7:**
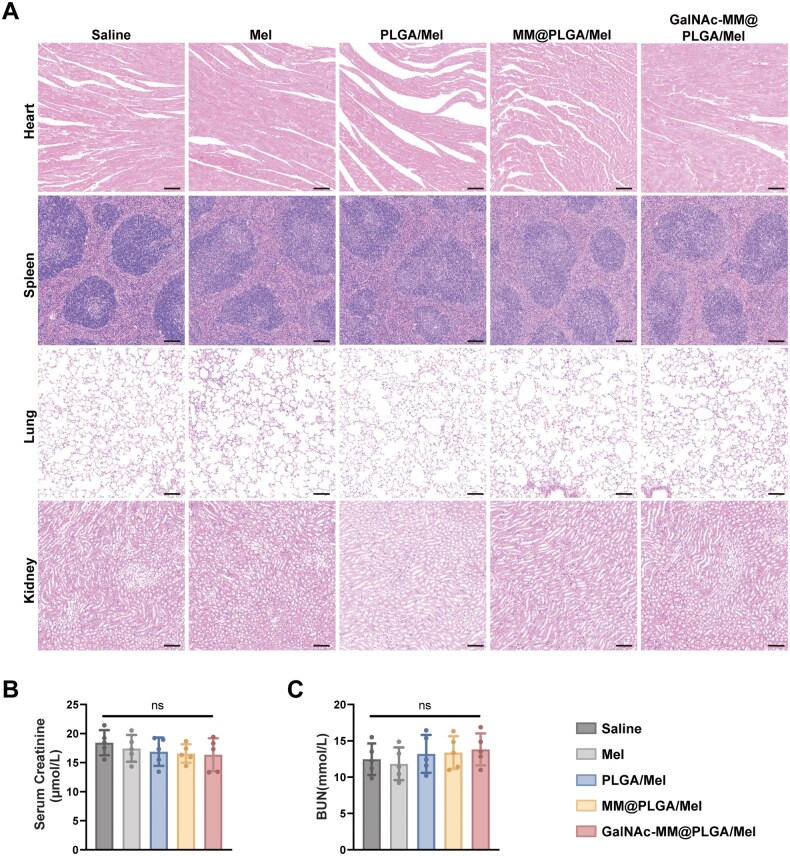
GalNAc-MM@PLGA/Mel exhibited good biosafety. (**A**) Results of HE staining in the murine spleen, heart, lungs and kidneys from different treatments (scale bar, 100 μm). (**B** and **C**) Blood biochemical test results, including the levels of serum BUN and creatinine (*n* = 5, ‘ns’ indicates no significance).

This study provided a comprehensive assessment of the biosafety of GalNAc-MM@PLGA NPs. At the conclusion of the treatment, the heart, spleen, lungs and kidneys were harvested from different murine model and evaluated for potential toxicological effects through histological analysis. As depicted in Figure 7A, H&E staining demonstrated no evident alterations in tissue morphology or structure, and no notable damage or abnormalities were observed. Furthermore, assessment of renal function indicators, including serum creatinine and blood urea nitrogen (BUN), showed no signs of systemic toxicity among the different treatment groups, with no statistically significant differences observed (Figure 7B and C). Subsequently, we evaluated the long-term toxicity and material immunogenicity of GalNAc-MM@PLGA/Mel. Healthy C57BL/6J mice received intravenous injections of GalNAc-MM@PLGA/Mel on days 1, 3, 7, 14 and 28, while mice in the control group were administered normal saline. At the end of the experiment, blood samples and major organs were collected for further analysis. H&E staining of the major organs revealed almost no obvious histopathological abnormalities (Supplementary Figure S8A). The results of serum biochemical assays and routine hematological examinations showed no significant differences between the groups at any time point (Supplementary Figure S8B and C). Our results suggest that PLGA NPs coated with engineered macrophage membranes possess an excellent safety profile *in vivo*. Overall, these results highlight the bio-compatibility of the NPs and emphasize their potential applicability in future biomedical applications.

## Conclusion

In this work, we highlighted the crucial involvement of ERS pathways in the inflammatory response associated with NAFLD and identified their cellular origin through bioinformatics analysis. Our findings demonstrated that Mel mitigated ERS pathways in hepatocytes, thereby reducing the secretion of inflammatory factors. Building upon these findings, we engineered GalNAc-MM@PLGA/Mel as a novel platform for treating NAFLD. These NPs maintained the immune-evasive features of previously established macrophage membrane-coated NPs, while the GalNAc modification enhances their targeting specificity to hepatocytes. As a result, these NPs successfully suppressed the activation of the ERS in hepatocytes by delivering Mel, leading to downregulation of NF-κB signaling pathways. In summary, these engineered, macrophage membrane-coated NPs demonstrated significant anti-inflammatory and lipid-lowering impacts via the inhibition of the ERS and its downstream NF-κB signaling pathway. Hence, the engineered cell NP-based therapies formulated in our research could represent a reliable, potent and economically viable approach for treating NAFLD.

## Supplementary Material

rbag084_Supplementary_Data

## Data Availability

Data can be obtained by contacting the corresponding author with a reasonable justification.

## References

[1] Younossi ZM , Golabi P, Paik JM, Henry A, Van Dongen C, Henry L. The global epidemiology of nonalcoholic fatty liver disease (NAFLD) and nonalcoholic steatohepatitis (NASH): a systematic review. Hepatology 2023;77:1335–47. 36626630 10.1097/HEP.0000000000000004 PMC10026948

[2] Huang DQ , El-Serag HB, Loomba R. Global epidemiology of NAFLD-related HCC: trends, predictions, risk factors and prevention. Nat Rev Gastroenterol Hepatol 2021;18:223–38. 33349658 10.1038/s41575-020-00381-6 PMC8016738

[3] Kim JY , Garcia-Carbonell R, Yamachika S, Zhao P, Dhar D, Loomba R, Kaufman RJ, Saltiel AR, Karin M. ER stress drives lipogenesis and steatohepatitis via caspase-2 activation of S1P. Cell 2018;175:133–45.e15. 30220454 10.1016/j.cell.2018.08.020 PMC6159928

[4] Wang M , Kaufman RJ. Protein misfolding in the endoplasmic reticulum as a conduit to human disease. Nature 2016;529:326–35. 26791723 10.1038/nature17041

[5] Puri P , Mirshahi F, Cheung O, Natarajan R, Maher JW, Kellum JM, Sanyal AJ. Activation and dysregulation of the unfolded protein response in nonalcoholic fatty liver disease. Gastroenterology 2008;134:568–76. 18082745 10.1053/j.gastro.2007.10.039

[6] Li W , Cao T, Luo C, Cai J, Zhou X, Xiao X, Liu S. Crosstalk between ER stress, NLRP3 inflammasome, and inflammation. Appl Microbiol Biotechnol 2020;104:6129–40. 32447438 10.1007/s00253-020-10614-y

[7] Rotman Y , Sanyal AJ. Current and upcoming pharmacotherapy for non-alcoholic fatty liver disease. Gut 2017;66:180–90. 27646933 10.1136/gutjnl-2016-312431

[8] Hagström H , Kechagias S, Ekstedt M. Risk for hepatic and extra-hepatic outcomes in nonalcoholic fatty liver disease. J Intern Med 2022;292:177–89. 34118091 10.1111/joim.13343

[9] Sun H , Huang F, Qu S. Melatonin: a potential intervention for hepatic steatosis. Lipids Health Dis 2015;14:75. 26199093 10.1186/s12944-015-0081-7 PMC4511016

[10] Pan M , Song Y, Xu J, Gan H. Melatonin ameliorates nonalcoholic fatty liver induced by high-fat diet in rats. J Pineal Res 2006;41:79–84. 16842545 10.1111/j.1600-079X.2006.00346.x

[11] Redza-Dutordoir M , Bates DAA. Activation of apoptosis signalling pathways by reactive oxygen species. Biochim Biophys Acta 2016;1863:2977–92. 27646922 10.1016/j.bbamcr.2016.09.012

[12] Yao M , Pu P, Li Z, Zhu K, Zhou L, Sun Y, Dai Y, Cui X, Wang Y. Melatonin restores endoplasmic reticulum homeostasis to protect injured neurons in a rat model of chronic cervical cord compression. J Pineal Res 2023;74:e12859. 36732085 10.1111/jpi.12859

[13] Reiter RJ , Tan DX, Galano A. Melatonin: exceeding expectations. Physiology (Bethesda) 2014;29:325–33. 25180262 10.1152/physiol.00011.2014

[14] Migni A , Bartolini D, Marcantonini G, Sardella R, Rende M, Tognoloni A, Ceccarini MR, Galli F. Melatonin repairs the lipidome of human hepatocytes exposed to Cd and free fatty acid-induced lipotoxicity. J Pineal Res 2025;77:e70047. 40193217 10.1111/jpi.70047 PMC11975211

[15] Cheung RTF. The utility of melatonin in reducing cerebral damage resulting from ischemia and reperfusion. J Pineal Res 2003;34:153–60. 12614473 10.1034/j.1600-079x.2003.00034.x

[16] Liu Z , Ran Y, Qie S, Gong W, Gao F, Ding Z, Xi J. Melatonin protects against ischemic stroke by modulating microglia/macrophage polarization toward anti-inflammatory phenotype through STAT3 pathway. CNS Neurosci Ther 2019;25:1353–62. 31793209 10.1111/cns.13261 PMC6887673

[17] Abulateefeh SR. Long-acting injectable PLGA/PLA depots for leuprolide acetate: successful translation from bench to clinic. Drug Deliv Transl Res 2023;13:520–30. 35976565 10.1007/s13346-022-01228-0

[18] Luo Z , Lu Y, Shi Y, Jiang M, Shan X, Li X, Zhang J, Qin B, Liu X, Guo X, Huang J, Liu Y, Wang S, Li Q, Luo L, You J. Neutrophil hitchhiking for drug delivery to the bone marrow. Nat Nanotechnol 2023;18:647–56. 37081080 10.1038/s41565-023-01374-7

[19] Liang H , Yan Y, Sun W, Ma X, Su Z, Liu Z, Chen Y, Yu B. Preparation of melatonin-loaded nanoparticles with targeting and sustained release function and their application in osteoarthritis. Int J Mol Sci 2023;24:8740. 37240086 10.3390/ijms24108740 PMC10217911

[20] Hu CJ , Zhang L, Aryal S, Cheung C, Fang RH, Zhang L. Erythrocyte membrane-camouflaged polymeric nanoparticles as a biomimetic delivery platform. Proc Natl Acad Sci U S A 2011;108:10980–5. 21690347 10.1073/pnas.1106634108 PMC3131364

[21] Yang L , Zang G, Li J, Li X, Li Y, Zhao Y. Cell-derived biomimetic nanoparticles as a novel drug delivery system for atherosclerosis: predecessors and perspectives. Regen Biomater 2020;7:349–58. 32793380 10.1093/rb/rbaa019 PMC7414994

[22] Rao L , Bu L, Xu J, Cai B, Yu G, Yu X, He Z, Huang Q, Li A, Guo S, Zhang W, Liu W, Sun Z, Wang H, Wang T, Zhao X. Red blood cell membrane as a biomimetic nanocoating for prolonged circulation time and reduced accelerated blood clearance. Small 2015;11:6225–36. 26488923 10.1002/smll.201502388

[23] Hou X , Zeng H, Chi X, Hu X. Pathogen receptor membrane-coating facet structures boost nanomaterial immune escape and antibacterial performance. Nano Lett 2021;21:9966–75. 34812644 10.1021/acs.nanolett.1c03427

[24] Duan Y , Zhou J, Zhou Z, Zhang E, Yu Y, Krishnan N, Silva-Ayala D, Fang RH, Griffiths A, Gao W, Zhang L. Extending the in vivo residence time of macrophage membrane-coated nanoparticles through genetic modification. Small 2023;19:e2305551. 37635117 10.1002/smll.202305551

[25] Li J , Qi J, Tang Y, Liu H, Zhou K, Dai Z, Yuan L, Sun C. A nanodrug system overexpressed circRNA_0001805 alleviates nonalcoholic fatty liver disease via miR-106a-5p/miR-320a and ABCA1/CPT1 axis. J Nanobiotechnology 2021;19:363. 34789275 10.1186/s12951-021-01108-8 PMC8596892

[26] Zhang J , Yang W, Zhu Y, Li Z, Zheng Y, Zhang Y, Gao W, Zhang X, Wu Z, Gao L. Microenvironment-induced programmable nanotherapeutics restore mitochondrial dysfunction for the amelioration of non-alcoholic fatty liver disease. Acta Biomater 2025;194:323–35. 39805524 10.1016/j.actbio.2025.01.019

[27] Qu Y , Chu B, Li J, Deng H, Niu T, Qian Z. Macrophage-biomimetic nanoplatform-based therapy for inflammation-associated diseases. Small Methods 2024;8:e2301178. 38037521 10.1002/smtd.202301178

[28] Krishnan N , Jiang Y, Zhou J, Mohapatra A, Peng F, Duan Y, Holay M, Chekuri S, Guo Z, Gao W, Fang RH, Zhang L. A modular approach to enhancing cell membrane-coated nanoparticle functionality using genetic engineering. Nat Nanotechnol 2024;19:345–53. 37903891 10.1038/s41565-023-01533-w PMC10954421

[29] Gu C , Geng X, Wu Y, Dai Y, Zeng J, Wang Z, Fang H, Sun Y, Chen X. Engineered macrophage membrane-coated nanoparticles with enhanced CCR2 expression promote spinal cord injury repair by suppressing neuroinflammation and neuronal death. Small 2024;20:e2305659. 37884477 10.1002/smll.202305659

[30] He X , Chang Z, Chen F, Zhang W, Sun M, Shi T, Liu J, Chen P, Zhang K, Guan S, Zhao Z, Li M, Dong W, Shao D, Yang C. Engineering a biomimetic system for hepatocyte-specific RNAi treatment of non-alcoholic fatty liver disease. Acta Biomater 2024;174:281–96. 37951519 10.1016/j.actbio.2023.10.038

[31] Simon-Szabó L , Kokas M, Mandl J, Kéri G, Csala M. Metformin attenuates palmitate-induced endoplasmic reticulum stress, serine phosphorylation of IRS-1 and apoptosis in rat insulinoma cells. PLoS One 2014;9:e97868. 24896641 10.1371/journal.pone.0097868 PMC4045581

[32] Nasiri-Ansari N , Nikolopoulou C, Papoutsi K, Kyrou I, Mantzoros CS, Kyriakopoulos G, Chatzigeorgiou A, Kalotychou V, Randeva MS, Chatha K, Kontzoglou K, Kaltsas G, Papavassiliou AG, Randeva HS, Kassi E. Empagliflozin attenuates non-alcoholic fatty liver disease (NAFLD) in high fat diet fed ApoE(−/−) mice by activating autophagy and reducing ER stress and apoptosis. Int J Mol Sci 2021;22:818. 33467546 10.3390/ijms22020818 PMC7829901

[33] Park NW , Lee ES, Ha KB, Jo SH, Kim HM, Kwon M, Chung CH. Umbelliferone ameliorates hepatic steatosis and lipid-induced ER stress in high-fat diet-induced obese mice. Yonsei Med J 2023;64:243–50. 36996895 10.3349/ymj.2022.0354 PMC10067795

[34] Das N , Mandala A, Naaz S, Giri S, Jain M, Bandyopadhyay D, Reiter RJ, Roy SS. Melatonin protects against lipid-induced mitochondrial dysfunction in hepatocytes and inhibits stellate cell activation during hepatic fibrosis in mice. J Pineal Res 2017;62:4. 10.1111/jpi.12404 28247434

[35] Shieh J , Wu H, Cheng K, Cheng J. Melatonin ameliorates high fat diet-induced diabetes and stimulates glycogen synthesis via a PKCzeta-Akt-GSK3beta pathway in hepatic cells. J Pineal Res 2009;47:339–44. 19817973 10.1111/j.1600-079X.2009.00720.x

[36] Stacchiotti A , Favero G, Lavazza A, Golic I, Aleksic M, Korac A, Rodella L F, Rezzani R. Hepatic macrosteatosis is partially converted to microsteatosis by melatonin supplementation in Ob/ob mice non-alcoholic fatty liver disease. Plos One 2016;11:e148115. 10.1371/journal.pone.0148115 PMC4732686 26824477

[37] Stacchiotti A , Grossi I, Garcia-Gomez R, Patel GA, Salvi A, Lavazza A, De Petro G, Monsalve M, Rezzani R. Melatonin effects on non-alcoholic fatty liver disease are related to MicroRNA-34a-5p/Sirt1 axis and autophagy. Cells 2019;8:1053. 31500354 10.3390/cells8091053 PMC6770964

[38] Saha M , Manna K, Das Saha K. Melatonin suppresses NLRP3 inflammasome activation via TLR4/NF-κB and P2X7R signaling in high-fat diet-induced murine NASH model. J Inflamm Res 2022;15:3235–58. 35668917 10.2147/JIR.S343236 PMC9166960

[39] Guan Q , Wang Z, Hu K, Cao J, Dong Y, Chen Y. Melatonin ameliorates hepatic ferroptosis in NAFLD by inhibiting ER stress via the MT2/cAMP/PKA/IRE1 signaling pathway. Int J Biol Sci 2023;19:3937–50. 37564204 10.7150/ijbs.85883 PMC10411470

[40] D’Souza AA , Devarajan PV. Asialoglycoprotein receptor mediated hepatocyte targeting—strategies and applications. J Control Release 2015;203:126–39. 25701309 10.1016/j.jconrel.2015.02.022

[41] Lu X , Fu Y, Zhu Y, Xi C, Luo Q, Pang H. Construction of in-situ self-assembled agent for NIR/PET dual-modal imaging and photodynamic therapy for hepatocellular cancer. J Nanobiotechnology 2024;22:614. 39385303 10.1186/s12951-024-02879-6 PMC11465773

[42] Blanco E , Shen H, Ferrari M. Principles of nanoparticle design for overcoming biological barriers to drug delivery. Nat Biotechnol 2015;33:941–51. 26348965 10.1038/nbt.3330 PMC4978509

[43] Barua S , Mitragotri S. Challenges associated with penetration of nanoparticles across cell and tissue barriers: a review of current status and future prospects. Nano Today 2014;9:223–43. 25132862 10.1016/j.nantod.2014.04.008 PMC4129396

[44] Zhou Y , Deng Y, Liu Z, Yin M, Hou M, Zhao Z, Zhou X, Yin L. Cytokine-scavenging nanodecoys reconstruct osteoclast/osteoblast balance toward the treatment of postmenopausal osteoporosis. Sci Adv 2021;7:eabl6432. 34818042 10.1126/sciadv.abl6432 PMC8612675

[45] Xuan M , Shao J, Dai L, He Q, Li J. Macrophage cell membrane camouflaged mesoporous silica nanocapsules for in vivo cancer therapy. Adv Healthc Mater 2015;4:1645–52. 25960053 10.1002/adhm.201500129

[46] Chen T , Meng Y, Zhou Z, Li H, Wan L, Kang A, Guo W, Ren K, Song X, Chen Y, Zhao W. GAS5 protects against nonalcoholic fatty liver disease via miR-28a-5p/MARCH7/NLRP3 axis-mediated pyroptosis. Cell Death Differ 2023;30:1829–48. 37337032 10.1038/s41418-023-01183-4 PMC10307850

[47] Liu C , Zheng X, Ji J, Zhu X, Liu X, Liu H, Guo L, Ye K, Zhang S, Xu Y, Sun X, Zhou W, Wong HLX, Tian Y, Qian H. The carotenoid torularhodin alleviates NAFLD by promoting *Akkermanisa muniniphila*-mediated adenosylcobalamin metabolism. Nat Commun 2025;16:3338. 40199868 10.1038/s41467-025-58500-3 PMC11978934

[48] Hossain S , Gilani A, Pascale J, Villegas E, Diegisser D, Agostinucci K, Kulaprathazhe M, Dirice E, Garcia V, Schwartzman ML. Gpr75-deficient mice are protected from high-fat diet-induced obesity. Obesity (Silver Spring) 2023;31:1024–37. 36854900 10.1002/oby.23692 PMC10033368

[49] Colletti LM , Remick DG, Burtch GD, Kunkel SL, Strieter RM, Campbell DA. Role of tumor necrosis factor-alpha in the pathophysiologic alterations after hepatic ischemia/reperfusion injury in the rat. J Clin Invest 1990;85:1936–43. 2161433 10.1172/JCI114656 PMC296661

[50] Pan J , Fallon MB. Gender and racial differences in nonalcoholic fatty liver disease. World J Hepatol 2014;6:274–83. 24868321 10.4254/wjh.v6.i5.274 PMC4033285

[51] Lee C , Kim J, Jung Y. Potential therapeutic application of estrogen in gender disparity of nonalcoholic fatty liver disease/nonalcoholic steatohepatitis. Cells 2019;8:1259. 31619023 10.3390/cells8101259 PMC6835656

