# Supplementary material for: Cell membrane-coated nanoparticles against non-alcoholic fatty liver disease via regulating endoplasmic reticulum stress
Source: Regen Biomater. 2026 Apr 30;13:rbag084. doi: 10.1093/rb/rbag084 (PMC13202448; doi:10.1093/rb/rbag084)
Supplement: rbag084_Supplementary_Data [file rbag084_supplementary_data.docx]

Experimental Section

*Public data collection and differential gene analysis:* The transcriptome data were obtained from the Gene Expression Omnibus (GEO) database for Gene Ontology (GO) enrichment analysis and Gene Set Enrichment Analysis (GSEA). The specific dataset used was GSE122660. To identify differentially expressed genes, we performed the Wilcoxon test, which screened for genes with at least a 4-fold difference in expression between the experimental and control groups (log2 Fold Change = 2). P<0.05 indicates a significant difference, meaning the resulting genes are considered differentially expressed genes. The single-cell sequencing data used were from dataset GSE232182, and the data for gene correlation analysis were sourced from GSE167523. The results were visualized using the website (https://cloud.oebiotech.com/), while the remaining data processing was conducted in R (version 4.2.1).

*Characterization of the Physicochemical Properties of Nanoparticles:* The particle size distribution and zeta potential of nanoparticles (NPs) were measured using a NanoBrook instrument (Brookhaven Instruments, USA). The morphology of the NPs was examined with a transmission electron microscope (Hitachi, Japan). The NPs were incubated in DMEM supplemented with 10% fetal bovine serum at room temperature for various durations to evaluate their stability in serum. The levels of membrane proteins in PLGA, MM@PLGA, GalNAc-MM@PLGA, RAW264.7 cells, and MM were detected using Coomassie blue staining.

*Drug loading and in vitro drug release study:* The PLGA/Mel, MM@PLGA/Mel, and GalNAc-MM@PLGA/Mel NPs were first frozen at -80 °C and then lyophilized using a Labconco FreeZone lyophilizer. The lyophilized nanoparticle powders were subsequently dissolved in DMSO, and the absorbance was measured at 222 nm using a UV-visible spectrophotometer (DU730, Beckman Coulter). Using the standard curve of Mel in DMSO, we calculated the drug loading and encapsulation rates as shown below:

$$\text{loading content}\left( \text{\%} \right)\text{=}\frac{\text{M}_{\text{M}\text{el}}}{\text{M}_{\text{P}\text{LGA}}\text{+}\text{M}_{\text{Mel}}}\text{×1}\text{00\%}$$

$$\text{encapsulation efficiency}\left( \text{\%} \right)\text{=}\frac{\text{M}_{\text{M}\text{el}}}{\text{M}_{\text{a}\text{dded}}}\text{×1}\text{00\%}$$

where *M_Mel_* is the mass of Mel loaded in the NPs, *M_PLGA_* is the mass of polymer in the formulation and *M_added_* is the mass of Mel added.

The release of the drug (Mel) from PLGA/Mel, MM@PLGA/Mel, and GalNAc-MM@PLGA/Mel NPs was studied using a dialysis method. Specifically, solutions of PLGA/Mel, MM@PLGA/Mel, and GalNAc-MM@PLGA/Mel NPs were placed into a disposable dialysis bag (3.5 kDa molecular weight cutoff [MWCO], Millipore) and immersed in 10 mL of phosphate-buffered saline (PBS) solution (release medium, pH 7.4) at 37°C. Three independent replicates were performed for each sample. At various time points, 1 mL of the release medium was collected for analysis, and it was replaced with an equal volume of fresh PBS at 37°C. The cumulative amount of released Mel was quantified using a UV/Vis spectrophotometer (DU730, Beckman Coulter) at a wavelength of 222 nm.

*Quantitative Reverse Transcription PCR (RT-qPCR) Assay:* Total RNA was extracted from treated cells and tissues using TRIzol (Absin) and subsequently reverse-transcribed into cDNA using the PrimeScript RT Kit (MCE). RT-qPCR was carried out using the SYBR Green qPCR Premix (MCE). The cycling conditions consisted of an initial step at 95°C for 1 minute, followed by 40 cycles, each cycle involving 15 seconds at 95°C and 45 seconds at 60°C. The results were summarized as follows: The relative expression of the target gene was calculated using β-actin as the internal reference gene. The primer sequences used in the assay were as follows:

TNF-α (forward: 5'-CCCTCACACTCACAAACCAC-3′. reverse: 5′-ACAAGGTACAACCCATCGGC-3′)

ATF4 (forward: 5'-ACTTGATGTCCCCCTTCGAC-3′. reverse: 5′-CGGAAAAGGCATCCTCCTT-3′)

ATF6 (forward: 5'-CCAGATGAAGACTGGGAGTCG-3′. reverse: 5′-TGTCTGAGCAGAAGTGGCTG-3′)

DDIT3 (forward: 5'-CCCCAGGAAACGAAGAGGAAG-3′. reverse: 5′-ATGTGCGTGTGACCTCTGTT-3′)

β-actin (forward: 5'-CCAGCCTTCCTTCTTGGGTAT-3′. reverse: 5′-TGGCATAGAGGTCTTTACGGA-3′)

*Cytotoxicity assay:* The CCK-8 assay was employed to evaluate cell viability in vitro. AML12 cells were seeded in 96-well culture plates at a density of 1 × 10^4^ cells per well. The adherent cells were co-incubated with various concentrations of the test materials for 48 hours. Cell viability was then assessed using the CCK-8 reagent (Beyotime). Absorbance values were recorded at 450 nm using an enzyme marker (varioskan LUX, Thermo Fisher Scientific).

*Isolation of Primary Hepatocytes:* In this procedure, the liver was digested in situ using sequential injections of calcium-free Hank's solution and IV collagenase solution through the portal vein of mice. The liver tissue was then isolated, sectioned, and further digested in vitro with the IV collagenase solution. Hepatocytes were obtained by filtering the digested tissue through a 70 μm sieve, followed by centrifugation at 50 × g for 5 minutes.

*Endocytosis assay:* To evaluate the impact of macrophage membrane camouflage on phagocytosis, we conducted experiments using RAW264.7 macrophages. We examined the internalization of PLGA/DiI, MM@PLGA/DiI, and GalNAc-MM@PLGA/DiI by these macrophages. In brief, RAW264.7 macrophages were seeded in culture plates at a density of 5 × 10^4^ cells per well and cultured for 24 hours. Following this, we added PLGA/DiI, MM@PLGA/DiI, and GalNAc-MM@PLGA/DiI (at a concentration of 100 µg/mL PLGA/DiI per well). After incubating for 0.5, 1, 2, and 4 hours, respectively, we measured the average fluorescence intensity of the cells using flow cytometry (CytoFLEX, Beckman).

Human Hepatocarcinoma Cells (HepG2) was inoculated into well plates at a density of 5 × 10⁴ cells per well and cultured for 24 hours. Subsequently, PLGA/DiI, MM@PLGA/DiI, and CXCR2-MM@PLGA/DiI (with 100 µg/ml of PLGA/DiI per well) were added. After a 4-hour incubation, the average fluorescence intensity of the cells was measured using flow cytometry.

Mouse primary hepatocytes, mouse hepatocarcinoma cells (Hepa1-6), and RAW264.7 were seeded into well plates at a density of 5 × 10⁴ cells per well and cultured for 24 hours. Following this incubation period, PLGA/DiI, MM@PLGA/DiI, and CXCR2-MM@PLGA/DiI (100 µg/ml PLGA/DiI per well) were added. After an additional 4-hour incubation, the average fluorescence intensity of the cells was assessed using flow cytometry.

*In vitro model construction of NAFLD:* AML12 cells were cultured in DMEM/F-12 medium (Gibco, New York, USA) supplemented with 10% fetal bovine serum, 1% penicillin-streptomycin dual antibody, 1% ITS additive, and 40 ng/mL dexamethasone (Sigma). The cells were maintained at 37°C in a 5% CO2 atmosphere. To create an in vitro model that simulates a high-fat environment, the cells were stimulated with oleic acid (OA, 660 μM) and palmitic acid (PA, 330 μM). The BSA solution was added as a control.

*Flow cytometry:* F4/80 and CD68 expression in RAW 264.7 cells, as well as in GalNAc-MM@PLGA and other samples, was assessed. In brief, the samples were incubated with an anti-mouse F4/80 antibody (PE, BioLegend, USA) and an anti-mouse CD68 antibody (APC, BioLegend, USA) for 30 minutes at 4°C, protected from light. After incubation, the samples were washed with PBS, centrifuged at 3000 rpm for 5 minutes at 4°C, and then analyzed using a flow cytometer (FACSVerse 8, BD, USA). For the flow assay of reactive oxygen species, cells in dishes were collected after group treatment, added with diluted DCFH-DA, incubated for an appropriate period of time to place under a flow cytometer for detection.

*Histological and Immunofluorescence Analyses:* Liver tissues were paraffin-embedded, then cut into sections approximately 8-10 μm thick and stained with hematoxylin and eosin (H&E). The expression of phosphorylated NF-κB (p-NF-κB) and tumor necrosis factor-alpha (TNF-α) was assessed through immunofluorescence. Briefly, after sealing the tissue sections, they were incubated overnight at 4°C with primary antibodies against p-NF-κB and TNF-α. Following washing with PBS, fluorescent secondary antibodies were applied and incubated at room temperature for 50 minutes, after which the sections were restained with DAPI. Images of the sections were captured using a fluorescence microscope (Nikon Ti2) and quantified with ImageJ. Additionally, immunohistochemistry (IHC) was conducted to detect the expressions of AFT6, AFT4, and CHOP. After closure, tissue sections were incubated overnight at 4°C with primary antibodies against AFT6, AFT4, and CHOP. Following PBS washes, fluorescent secondary antibodies were added and incubated for 50 minutes at room temperature. Color development was achieved using DAB (Solarbio), followed by staining with hematoxylin (Beyotime). Images of the sections were obtained with a light microscope (Leica) and quantified using ImageJ.

*Oil Red O Staining:* Fresh liver tissue was collected and embedded in Optimal Cutting Temperature (OCT) compound. It was then immediately snap-frozen in liquid nitrogen and sliced into 8-μm sections. The sections were fixed in 75% alcohol for 10 minutes. Following this, the tissue was treated with 60% isopropyl alcohol for 2 minutes and stained with Oil Red O for 8 minutes. Finally, the nuclei were stained with hematoxylin.

*In Vivo Imaging:* For in vivo imaging, C57BL/6 mice were randomly assigned to three groups: (1) PLGA/DiR group, (2) MM@PLGA/DiR group, and (3) GalNAc-MM@PLGA/DiR group. Each group received an equal number of NPs (2 mg/kg PLGA/DiR). The NPs were injected intravenously through the tail vein. Imaging of the in vivo distribution was conducted at various time points using an AniView Pro system (USA), with an excitation wavelength of 748 nm and an emission wavelength of 780 nm. Following the imaging, the mice were euthanized, and their major organs were extracted. The fluorescence intensity was measured in the heart, liver, spleen, kidneys, and lungs to evaluate the in vitro distribution of the NPs.

*Enzyme-Linked Immunosorbent Assay (ELISA)*: To evaluate the expression levels of TNF-α and IL-1β in mouse serum, serum samples were collected from mice belonging to different treatment groups. The concentrations of the cytokines were measured using ELISA kits (Jiubang Biotechnology, China). Optical density (OD) values were obtained using a Varioskan LUX enzyme marker (Thermo Fisher Scientific), and the cytokine concentrations were calculated based on the standard curve.

*In Vivo Toxicity Evaluation:* Toxicity assessments and in vivo histopathological studies were conducted following treatment. Organ specimens, including the heart, spleen, lungs, and kidneys, were collected from mice in the saline, PLGA/Mel, MM@PLGA/Mel, and GalNAc-MM@PLGA/Mel groups for sectioning. All specimens were subsequently stained with hematoxylin and eosin using the Hematoxylin and Eosin Staining Kit (Biyoung) and were examined histopathologically. Additionally, blood samples were collected from the different groups of experimental animals, and blood urea nitrogen (BUN) and serum creatinine levels were assessed using biochemical assays. C57BL/6J mice were randomly divided into five groups (n = 5) and sacrificed after receiving intravenous injections of GalNAc-MM@PLGA/Mel (1 mg/kg mel each group) for different time points (0, 1, 3, 7, 14, and 28 days). The blood of S15 each mouse was collected for routine blood examination and biochemistry assay. The major organs of the mice were also collected and stained with H&E.

*Statistical Analysis:* Statistical analysis of the experimental results was conducted using GraphPad Prism 9 software. The data are presented as the mean ± standard deviation (s.d.) and are based on at least three independent experiments (n ≥ 3). Comparisons between two groups were made using Student's t-test, with P-values less than 0.05 considered statistically significant. For comparisons involving multiple experimental groups, two-way ANOVA was employed.

**Supplementary Table S1.** Details on the antibodies used in the present study

| **Antibodies** | **Source** | **Dilution** | **Catalogue number** | **Manufacturer** |
| --- | --- | --- | --- | --- |
| Anti-P-IRE1 antibody | Rabbit | WB: 1:1500 | AP1442 | abclonal |
| Anti-P-PERK antibody | Rabbit | WB:1:1500 | AP1501 | abclonal |
| Anti-ATF6 antibody | Rabbit | WB:1:500 | ET1701 | Huabio |
| Anti-p-NF-κB antibody | Rabbit | WB: 1:500  IHC: 1:100 | AP0124 | abclonal |
| Anti-ATF4 antibody | Rabbit | WB: 1:1500 | ET1612 | Huabio |
| Anti-β-actin antibody | Mouse | WB: 1:1500 | 66009-1-Ig | Proteintech |
| Anti-CHOP antibody | Rabbit | WB: 1:500 | A20987 | abclonal |
| Anti-BIP antibody | Rabbit | WB: 1:1500  IHC:1:200 | ER1706 | Huabio |
| Anti-TNF-α antibody | Rabbit | WB: 1:1500 | 17590-1-AP | Proteintech |
| Anti-TNF-α antibody | Rabbit | IHC: 1:200 | GB115701 | [Servicebio](https://www.baidu.com/link?url=mqXL0JCE06yhgCXA8oRyqazqrlpgr-_mxtm-hnTS0q7&wd=&eqid=f40bddd0000145c60000000662383f86) |
| PE Anti-CD68 antibody | Rat | FC: 1:100 | 137013 | Biolegend |
| APC Anti-F4/80 antibody | Rat | FC: 1:100 | 2691600 | invitrogen |
| Anti-β-Tubulin antibody | Rabbit | WB: 1:2500 | R20005 | Abmart |
| HRP-conjugated Goat Anti-Rabbit IgG | Goat | WB: 1:5,000  IHC:1:1000 | D110058 | Sangon |
| HRP-conjugated Goat Anti-Mouse IgG | Goat | WB: 1:5,000 | D110087 | Sangon |


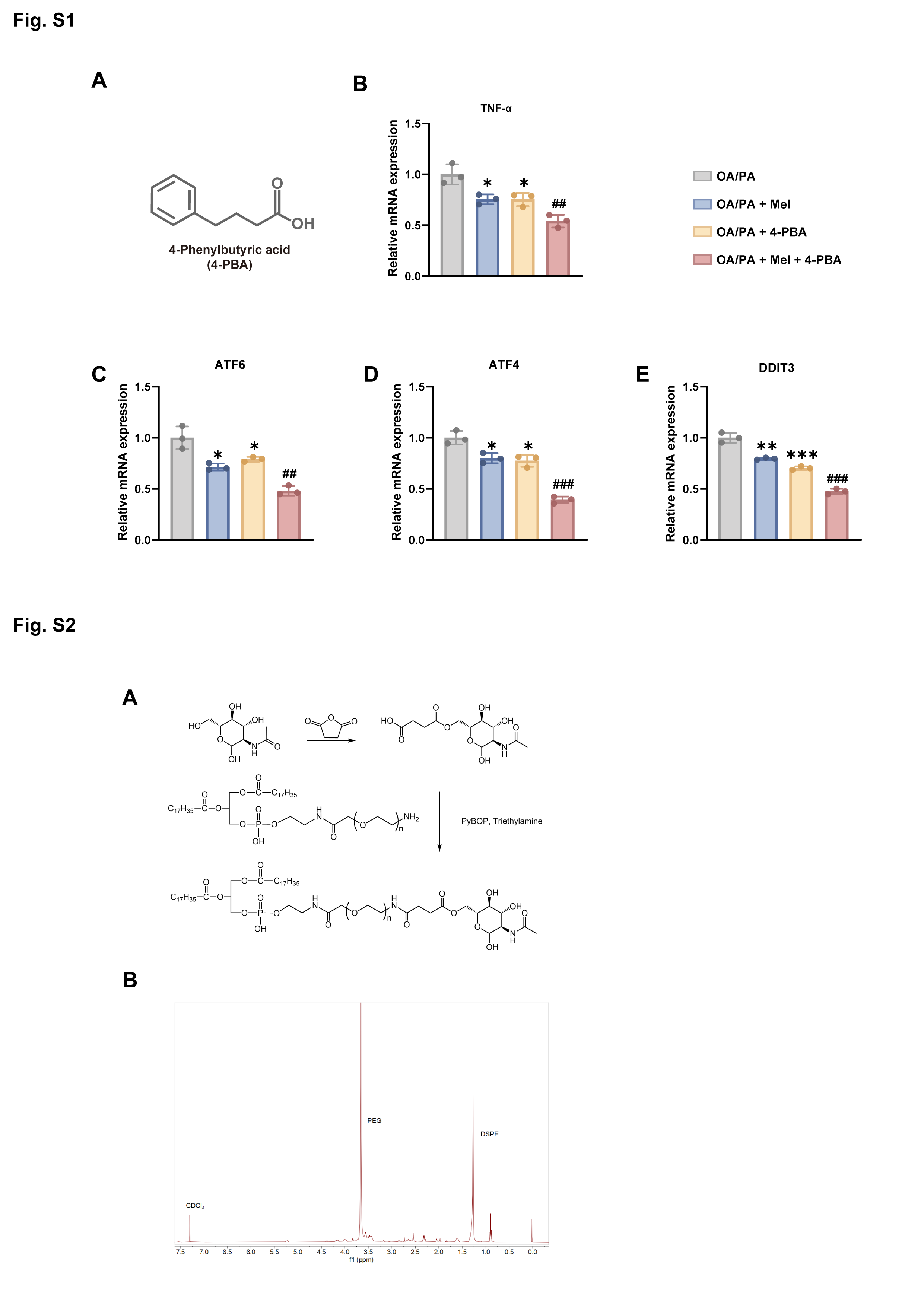


**Figure S1.** Relief of endoplasmic reticulum stress significantly reversed phenotype of AML12. A: 3D structure schematic of 4-PBA (4-PBA, 4-phenylbutyric acid). B: PCR results of inflammatory-related genes in AML12 after different treatments, with β-actin as a control. C-E: PCR results of endoplasmic reticulum stress-related genes in AML12 after different treatments, with β-actin as a control. (n = 3, *p < 0.05, **p < 0.01, ***p < 0.001, compared with OA/PA treatment group; ##p < 0.01, ###p < 0.001, compared with OA/PA+Mel treatment group).


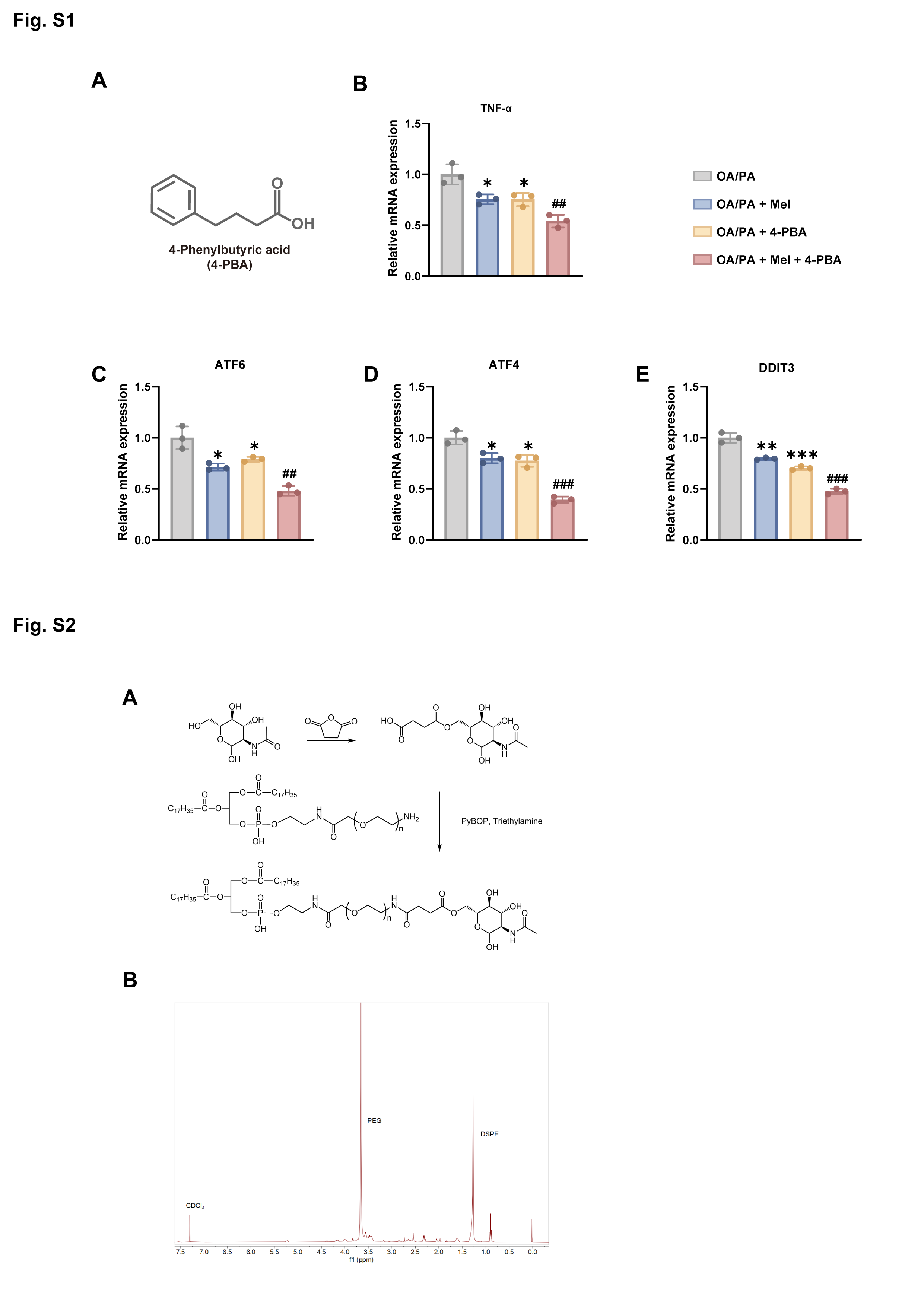


**Figure S2.** The synthesis of DSPE-PEG2000-GalNAc. A: structural route of DSPE-PEG2000-GalNAc. B: 1H NMR spectrum of DSPE-PEG2000-GalNAc.


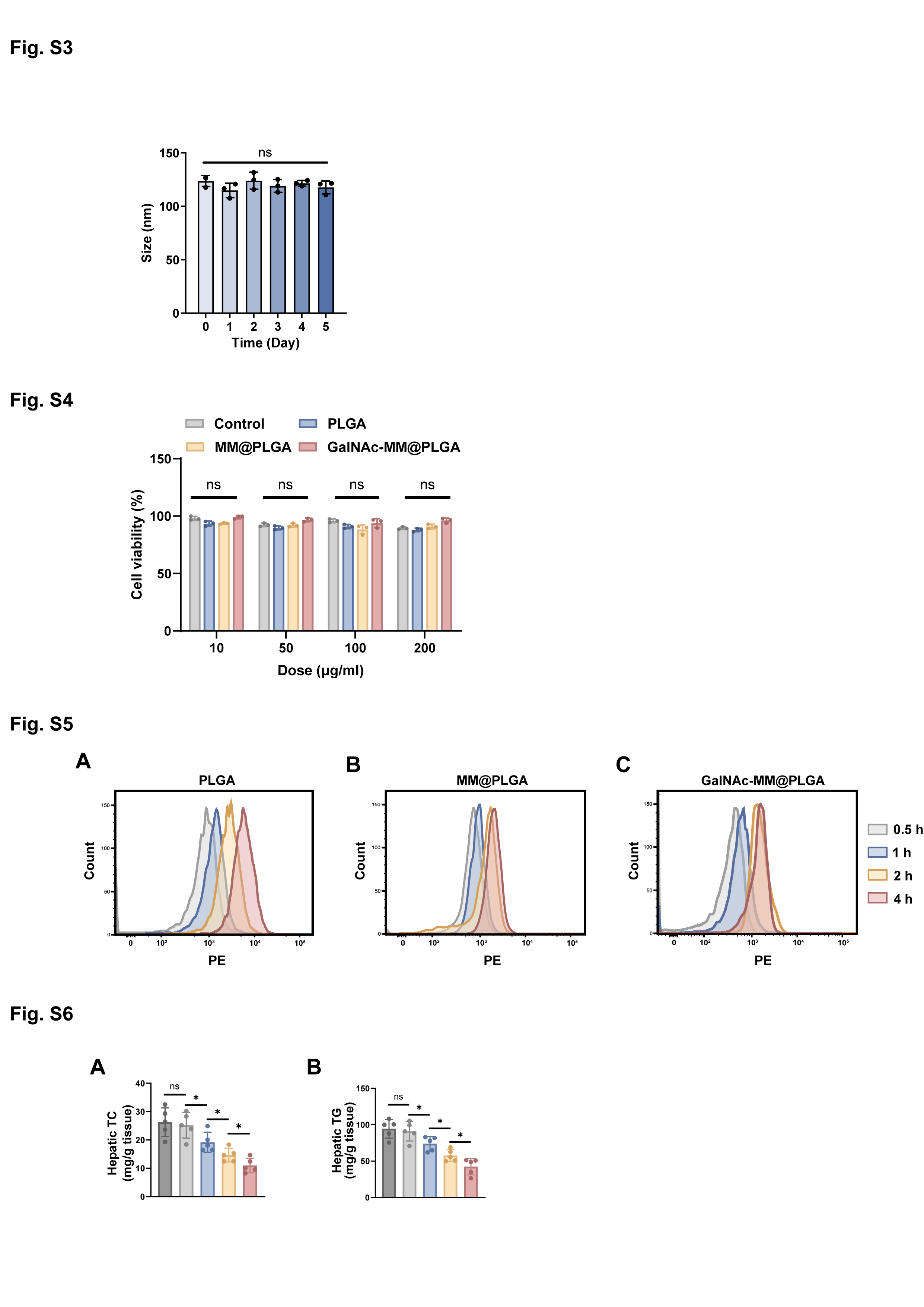


**Figure S3.** Mean diameter of GalNAc-MM@PLGA/Mel after 0, 1, 2, 3, 4, and 5 days of storage in medium containing 10% fetal bovine serum at room temperature. (n = 3, "ns" denotes no significance).


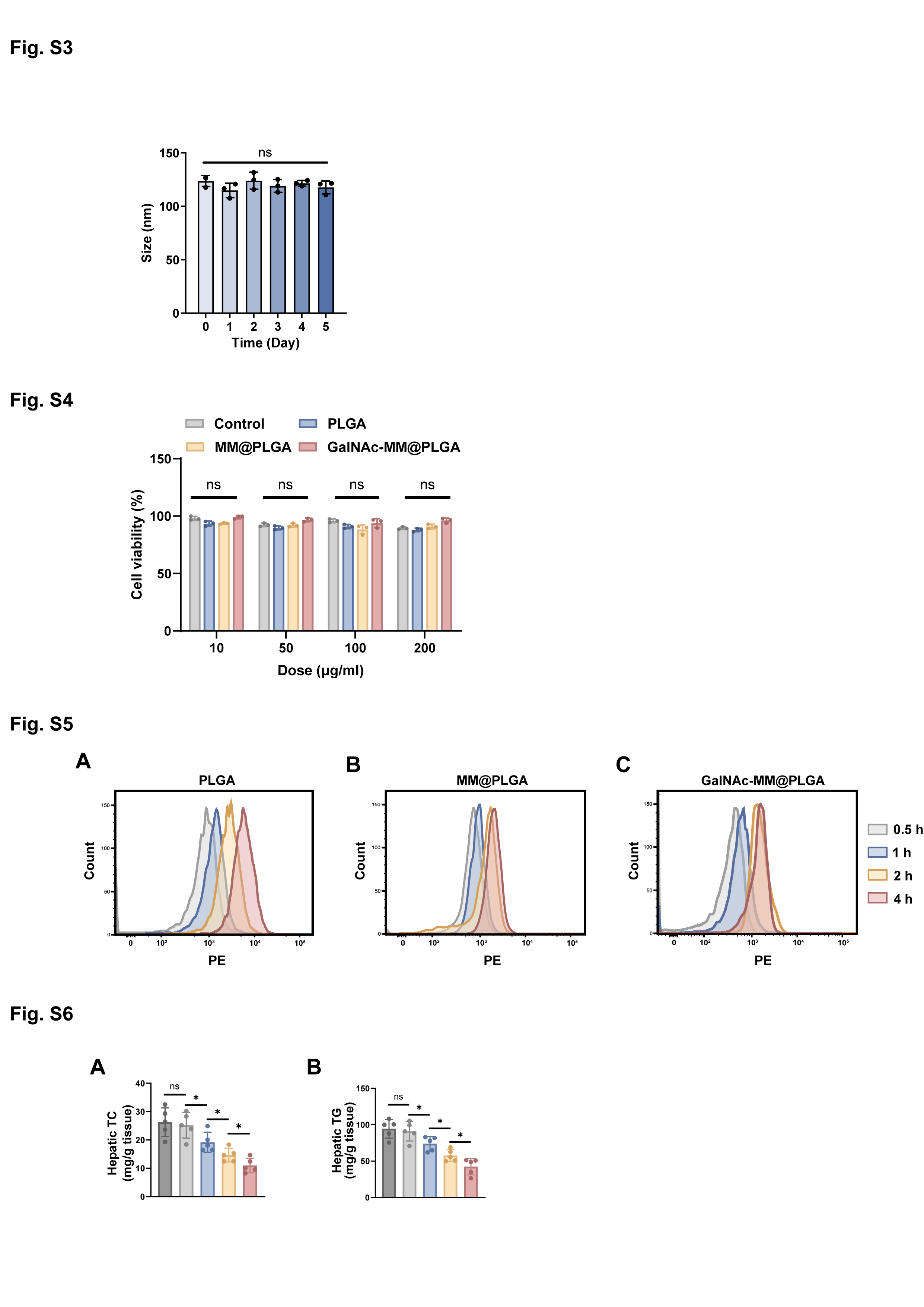


**Figure S4.** Cell survival of AML12 cells after 24 h of incubation with different concentrations of PLGA, MM@PLGA, and GalNAc-MM@PLGA NPs was assayed by CCK-8. (n = 3, "ns" denotes no significance).


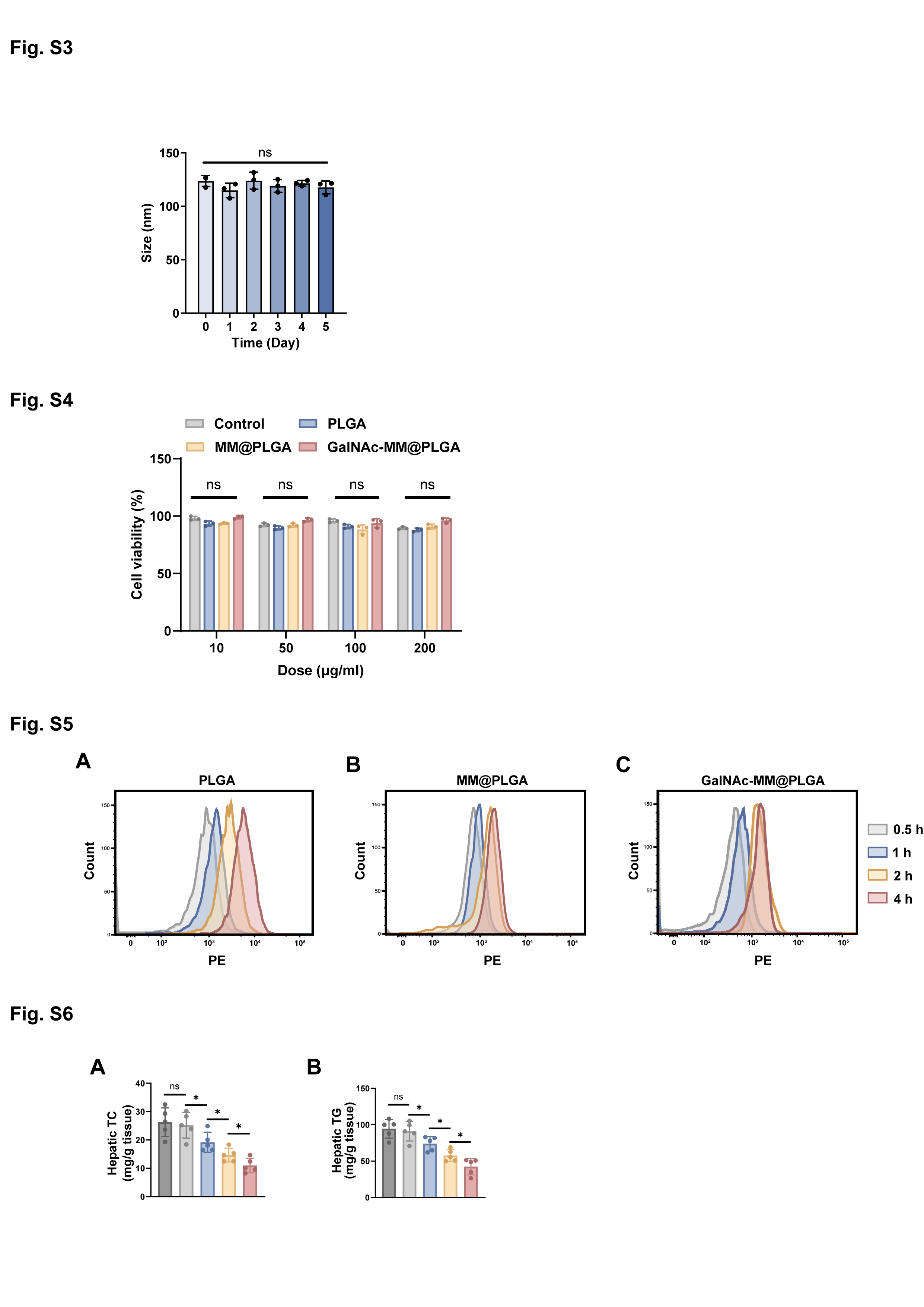


**Figure S5.** FACS results of cellular uptake of A: PLGA/DiI, B: MM@PLGA/DiI, and C: GalNAc-MM@PLGA/DiI in RAW264.7 cells.


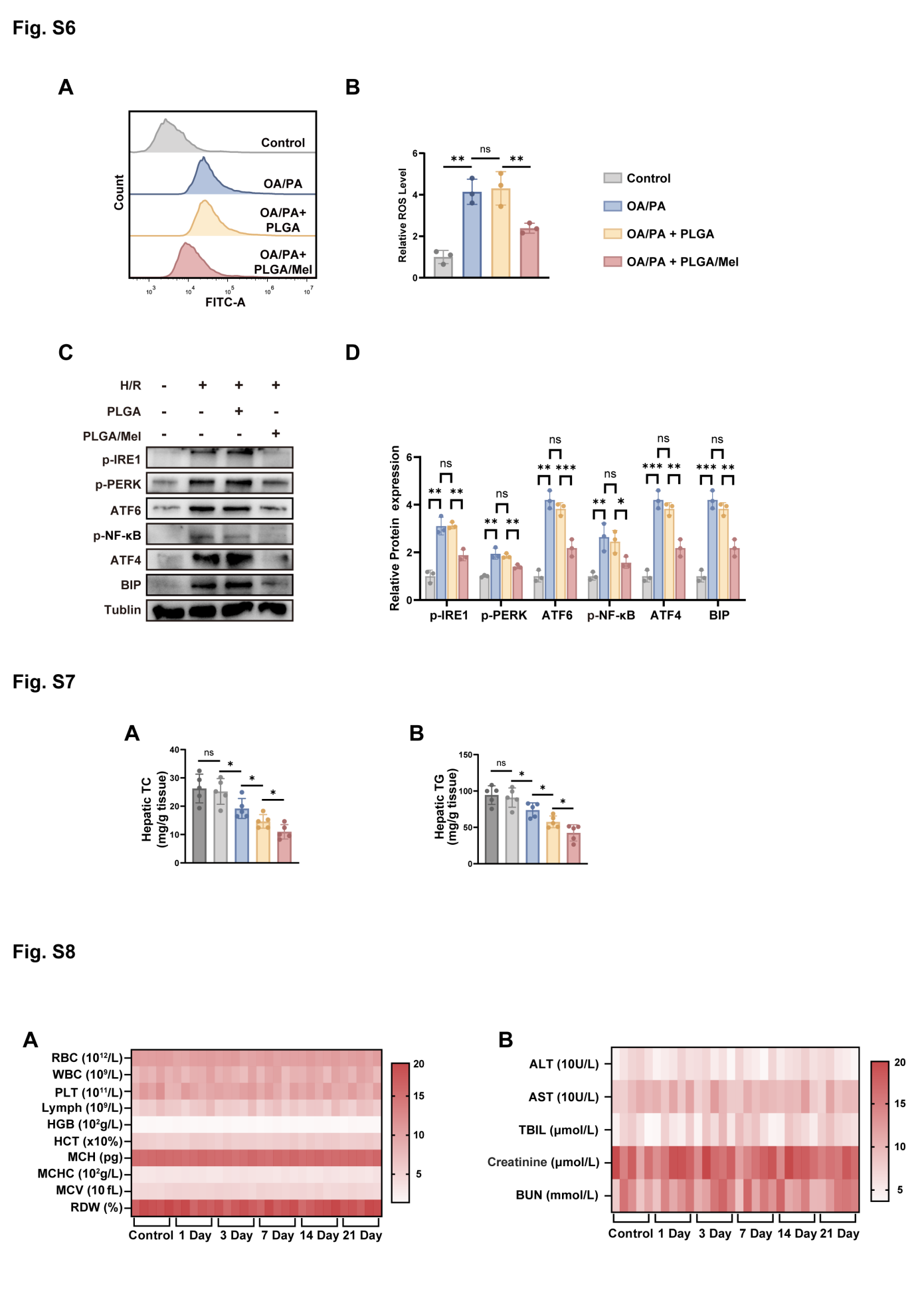


**Figure S6.** A-B: Flow analysis and quantitative analysis of the expression level of reactive oxygen species in AML-12 cells after different treatments. C-D: Western blot analysis of p-IRE1, p-PERK, p-NF-κB, ATF6, ATF4, and BIP expression in AML12 cells treated with OA/PA and PLGA or PLGA/Mel, showing their relative expression and quantification, with Tublin as a control. (n = 3, "ns" denotes no significance, *p < 0.05, **p < 0.01, ***p < 0.001).


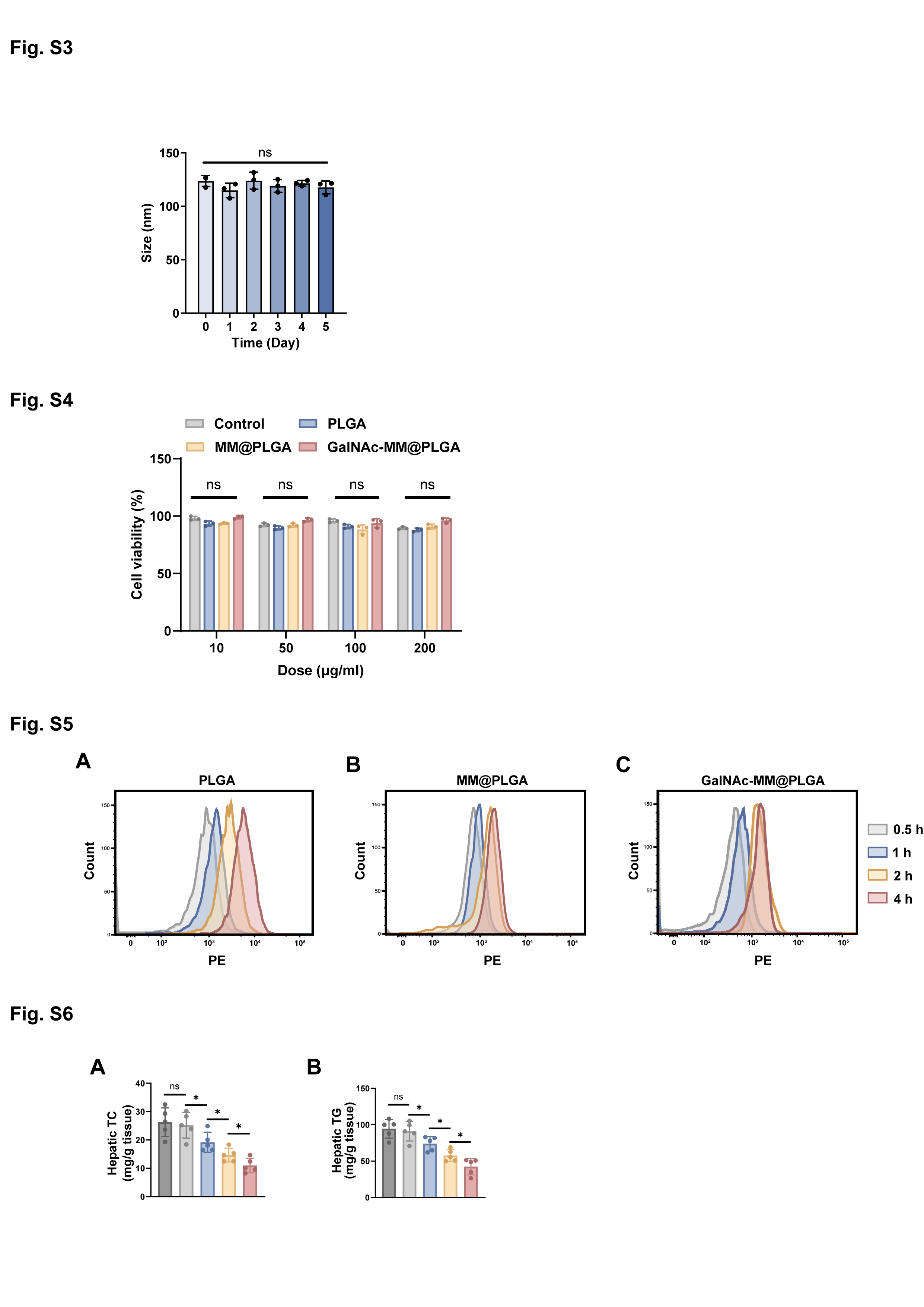


**Figure S7.** GalNAc-MM@PLGA NPs reduce lipid accumulation in liver tissue. A: Hepatic total cholesterol (TC) content from NAFLD mice treated with saline, Mel, PLGA/Mel, MM@PLGA/Mel, or GalNAc-MM@PLGA/Mel. B: Hepatic triglyceride (TG) content in different groups. (n = 5, "ns" denotes no significance, *p < 0.05).

**Figure S8.** A: H&E staining of the main organs (heart, liver, spleen, lung, and kidneys) in mice sacrificed at different time intervals (0 day, 1 day, 3 days, 7 days, 14 days, 28 days) after intravenous injection of GalNAc-MM@PLGA/Mel, the scale bars are 100 μm. B: Routine blood and C: blood biochemistry analysis of mice sacrificed on certain days after GalNAc-MM@PLGA/Mel treatment (n = 5).
